# Feasibility and scalability of a fitness tracker study: Results from a longitudinal analysis of persons with multiple sclerosis

**DOI:** 10.3389/fdgth.2023.1006932

**Published:** 2023-02-28

**Authors:** Chloé Sieber, Christina Haag, Ashley Polhemus, Ramona Sylvester, Jan Kool, Roman Gonzenbach, Viktor von Wyl

**Affiliations:** ^1^ Institute for Implementation Science in Health Care, Faculty of Medicine, University of Zürich, Zürich, Switzerland; ^2^ Epidemiology and Biostatistics and Prevention Institute, Faculty of Medicine, University of Zürich, Zürich, Switzerland; ^3^ Research Department Physiotherapy, Rehabilitation Centre, Valens, Switzerland

**Keywords:** mobile health (mHealth), multiple sclerosis, chronic disease, fitbit, wearable, adherence, scalability, lessons learned

## Abstract

**Background:**

Consumer-grade fitness trackers offer exciting opportunities to study persons with chronic diseases in greater detail and in their daily-life environment. However, attempts to bring fitness tracker measurement campaigns from tightly controlled clinical environments to home settings are often challenged by deteriorating study compliance or by organizational and resource limitations.

**Objectives:**

By revisiting the study design and patient-reported experiences of a partly remote study with fitness trackers (BarKA-MS study), we aimed to qualitatively explore the relationship between overall study compliance and scalability. On that account, we aimed to derive lessons learned on strengths, weaknesses, and technical challenges for the conduct of future studies.

**Methods:**

The two-phased BarKA-MS study employed Fitbit Inspire HR and electronic surveys to monitor physical activity in 45 people with multiple sclerosis in a rehabilitation setting and in their natural surroundings at home for up to 8 weeks. We examined and quantified the recruitment and compliance in terms of questionnaire completion and device wear time. Furthermore, we qualitatively evaluated experiences with devices according to participants' survey-collected reports. Finally, we reviewed the BarKA-MS study conduct characteristics for its scalability according to the Intervention Scalability Assessment Tool checklist.

**Results:**

Weekly electronic surveys completion reached 96%. On average, the Fitbit data revealed 99% and 97% valid wear days at the rehabilitation clinic and in the home setting, respectively. Positive experiences with the device were predominant: only 17% of the feedbacks had a negative connotation, mostly pertaining to perceived measurement inaccuracies. Twenty-five major topics and study characteristics relating to compliance were identified. They broadly fell into the three categories: “effectiveness of support measures”, “recruitment and compliance barriers”, and “technical challenges”. The scalability assessment revealed that the highly individualized support measures, which contributed greatly to the high study compliance, may face substantial scalability challenges due to the strong human involvement and limited potential for standardization.

**Conclusion:**

The personal interactions and highly individualized participant support positively influenced study compliance and retention. But the major human involvement in these support actions will pose scalability challenges due to resource limitations. Study conductors should anticipate this potential compliance-scalability trade-off already in the design phase.

## 1. Introduction

Mobile health (mHealth) describes the use of mobile devices, such as mobile phones and wearables, to collect health data to support and promote population wellness, but also for disease prevention, diagnosis, and management (1–4). Wearable devices such as consumer-grade fitness trackers offer continuous, passive, and inconspicuous collection of real-world data over a prolonged period of time (4, 5). Attractive key features of fitness trackers include the broad data collected by standard devices, ranging from physical activity (PA) levels and step counts to heart rate and sleep patterns (4, 6) as well as the high temporal measurement resolution. Therefore, such devices harbor great potential to facilitate a deeper understanding of complex disease expressions and phenotypes (5, 7).

In light of these potential advantages, there is a growing interest in using consumer-grade fitness trackers for health research (8), particularly in the field of multiple sclerosis (MS) (9, 10). Several characteristics of MS and its affected population lend themselves well as an interesting target for wearable device-based studies and disease management approaches. MS onset commonly occurs between 20 and 40 years of age, thus affecting age groups who are potentially well versed in electronic devices (9). Furthermore, the complex disease course of MS over decades with sometimes subtle but continuous symptom changes requires long-term continuous monitoring (11). A further hallmark feature of MS is the very heterogeneous symptom onset and presentation, which requires complex disease management strategies including different health care providers and treatment types (5, 12). Several very frequent symptoms such as gait impairment or fatigue are also suitable for monitoring with standard fitness trackers (13). In recent years, high-intensity PA has garnered attention as a potential means for improving health functioning and mitigating MS-related symptoms such as fatigue (14, 15).

However, consumer-wearables use in routine care settings at scale and over long time periods is still in its infancy (16–21), particularly in the domain of MS disease management (22). In the literature, the concept of “scalability” is defined as “deliberate efforts to increase the impact of successfully tested health interventions so as to benefit more people and to foster policy and program development on a lasting basis” (23). Scalability is a multifactorial concept and is influenced by numerous aspects that include the implementation context, evidence of effectiveness and cost-effectiveness, characteristics of the target population, as well as properties of the digital health tool or intervention to be implemented (24). These and other factors also form the foundation for the Intervention Scalability Assessment Tool (ISAT) tool that notably examines implementation and scale-up potential on five axes: (1) “fidelity and adaptation”, (2) “reach and acceptability”, (3) “delivery setting and workforce”, (4) “implementation infrastructure”, and (5) “sustainability” (24). The ISAT tool, along with similar other checklists (25), helps to assess the readiness interventions for a later scale-up.

In light of these scalability challenges, we developed the Barriers to physical activity in people with MS (BarKA-MS; https://clinicaltrials.gov/ct2/show/study/NCT04746807) fitness tracker study to explore barriers to PA among people with MS (PwMS) who returned home after an inpatient rehabilitation stay. The primary and secondary endpoints of the BarKA-MS study (analyzed elsewhere) explored common barriers to PA among PwMS and investigated the quality, reliability, internal consistency, and validity of PA metrics derived from a consumer-grade wearable device (26). A further analysis concerns the evaluation of the impact of inpatient rehabilitation on walking ability, PA and the perception of obstacles to PA, self-efficacy, fatigue, depression, pain, and health-related quality of life (Sieber et al., unpublished data, 2022).

The present analysis focuses on procedural aspects of the Barka-MS study and endeavors to provide a general assessment of the scalability of the BarKA-MS study design from the perspective of a later scale-up to a larger population and a longer follow-up duration. It aims to critically examine the scalability of key features of our BarKA-MS study by (1) analyzing study recruitment and factors associated with study recruitment and onboarding, (2) assessing study procedures adherence and data quality, (3) exploring participant usability experiences in wearing a consumer-grade fitness tracker, and (4) by deriving lessons learned and detecting room for improvement. These analyses used the ISAT scalability checklist for guidance (24).

## 2. Methods

### 2.1. The BarKA-MS study

The BarKA-MS study was an observational, longitudinal cohort study using consumer-grade fitness trackers, with the goal to monitor general PA during and after an inpatient rehabilitation stay among PwMS, as well as to identify PA barriers and facilitators (Table 1; https://clinicaltrials.gov/ct2/show/NCT04746807). This study was a collaboration between a research team from the University of Zurich, Switzerland, and the Kliniken Valens, a rehabilitation clinic specialized in neurological diseases located in Valens, Switzerland.

**Table 1:** Schedule of assessments throughout the BarKA-MS study.

Measures	Kliniken Valens					Home Environment			
	Week 0 (Baseline)	Week 1	Week 2	Week 3	Week 4	Week 5	Week 6	Week 7	Week 8
**Descriptive Measures**
Demographics (age, gender, living situation, home location)	X								
Health status (disease severity, time since diagnosis, relapse history, co-morbidities)	X								
**Physical Activity**
Self-report	X	X	X	X	X	X	X	X	X
Inspire-HR (worn daily)		X	X	X	X	X	X	X	X
Actigraph (worn daily)					X	X			
**Barriers to Physical Activity**
Barriers to Health Promoting Activities for Disabled Persons Scale	X				X				X
**Secondary Measures**
Depression	X				X				X
Walking ability	X				X				X
Fatigue	X				X				X
Health-related quality of life	X				X				X
Pain (visual analogue scales)	X				X				X
Self-efficacy	X				X				X
6 Min Walk Test^a^	X			X					
Timed Up and Go^a^	X			X					
10 Meter Walk Test^a^	X			X					
Weekly diary		X	X	X	X	X	X	X	X

^a^ Conducted routinely during inpatient rehabilitation at Valens. Assessment data analyzed within the BarKA-MS study.

The BarKA-MS study consisted of two phases (Table 1 and Supplementary Appendix Figure AS1): the first phase involved the recruitment and in-patient rehabilitation stay (1–4 weeks) of the study participants in the Kliniken Valens, and the second phase concerned the 4-weeks follow-up at the participants' home starting immediately after discharge. Sample size determination is available in the Supplementary Appendix (Methods Appendix – S1.2. Sample Size Determination for the BarKA-MS Study).

After successful recruitment including a signed written informed consent, study participants were invited to an introductory session with an on-site study coordinator from the Kliniken Valens. During this 1-hour session, the study coordinator provided the study participants with a Fitbit Inspire HR device, helped them install the corresponding Fitbit application on their phone, log in to their pre-configured and pseudonymized Fitbit account, and to pair the Fitbit tracker with the Fitbit application *via* Bluetooth. To minimize a co-intervention effect of the Fitbit device, alerts were turned off, the daily goals set to a minimum, and the app home screen was customized to only display sleep and heart rate. Nevertheless, step counts were still visible on the device screen and individuals had access to the Fitbit app.

Next, the on-site study coordinator created a participant account on the web-based *Research Management Information System* (RMIS) study survey platform (27), and completed the baseline questionnaire with the participant. Study participants received weekly invitations to a short survey, thus requiring them to access their emails *via* their mobile phone. A description of the survey instruments and physical capacity assessment tools is provided in the Supplementary methods of the Appendix (Methods Appendix – S1.3. Instruments).

During their rehabilitation stay, study participants had regular contact with and were supported by the on-site study coordinator. Once study participants returned to their home setting, the research team from the University of Zurich was available to provide remote support *via* emails, phone calls, and text messages. The study research team maintained logs of participant contacts and the technical or operational problems encountered during the study (hereafter “support log”).

### 2.2. Participants and recruitment

The BarKA-MS study aimed for a target recruitment goal of 45 participants. Study recruitment started in early January 2021 and ended at the end of September 2021. Data collection continued until mid-November 2021.

PwMS who were at Kliniken Valens for an in-patient rehabilitation stay were screened upon arrival and consecutively recruited by an on-site study coordinator. To be eligible for participation, these persons had to (1) be aged 18 years or older, (2) have a confirmed diagnosis of relapsing or progressive MS, (3) have an Expanded Disability Status Scale (EDSS) score of 2.0–6.5 (i.e., with reduced walking ability but are still able to walk independently with or without an assistive device), and not use a wheelchair at home, (4) be able to answer the surveys in German, (5) own a personal computer, a tablet or a mobile phone with Bluetooth and Wi-Fi functionalities, and (6) be willing to participate. Additional exclusion criteria were applied, namely, the inability to complete the baseline questionnaire, operate the consumer-grade wearable device and its application, or to engage safely in PA.

### 2.3. Wearable device measurements

In our observational study, Fitbit trackers were employed as an instrument to observe physical activity in real-life settings. They were not tied to or intended to act as an intervention. All participants received a Fitbit Inspire HR device. They were allowed to keep the device upon study completion, but no other incentive was provided. The metrics of interest monitored by this tracker were step count, PA intensity, and heart rate extracted in one-minute epochs. Additional metrics, such as energy expenditure, sleep duration and quality were also evaluated by this device. GPS functionality was deactivated by the study research team. Fitbit accounts were connected with Fitabase (Small Steps Labs LLC., CA. USA), a data management portal for studies using wearables. Study participants were asked to wear the Fitbit on their non-dominant wrist during the day for at least ten hours, and optionally during the night, throughout the study duration. A valid wear day corresponded to at least 10 h of wear per day between 6:00 a.m. and 11:00 p.m.

In addition, the study participant wore an Actigraph GT3X (Manufacturing Technology, Inc., FL, USA), a three-dimensional accelerometer validated for PwMS (28, 29), on their non-dominant hip during their last week of rehabilitation and the first week back home. These data were published elsewhere (26, 30).

### 2.4. Statistical analysis

Our analysis included eligible individuals who had completed the study (dropouts were not included). Device data from discharge days were excluded from the analyses.

Descriptive statistics were used for the characterization of the study participants and for the evaluation of the completeness of the collected data. Study characteristics included demographics, health, and additional baseline information (i.e., change in PA level, barriers to PA, PA level, walking ability, fatigue, self-efficacy, depression, general health, pain, walking endurance, walking speed, balance and dynamic functional mobility). Continuous data were analyzed by medians and interquartile ranges (IQR) and categorical information by frequency counts and percentages (%). The statistical analyses were conducted in R, version 4.0.3 (31) using the RStudio environment, version 1.4.1103 (32).

**Figure 1:**
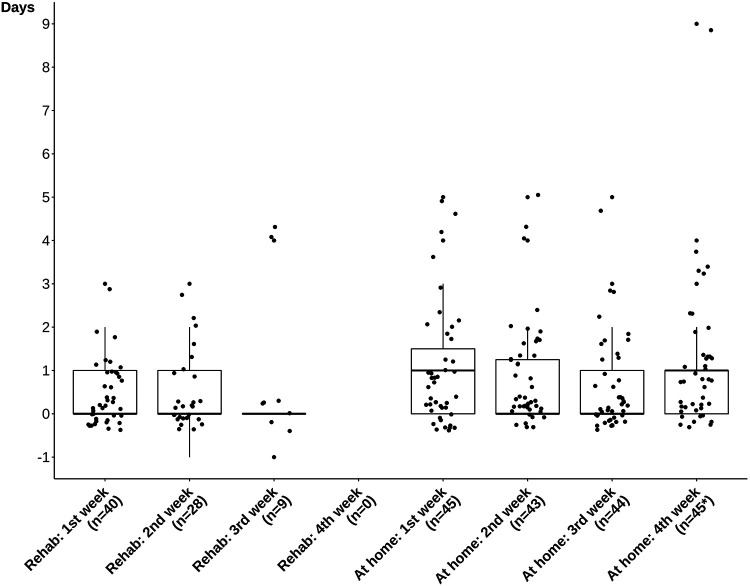
Time elapsed between the invitation and completion of the different surveys study participants had to complete on their own. The “baseline” and “end of rehabilitation” surveys were completed together with the person of contact in the rehabilitation clinic and are therefore not displayed. Due to technical issues, twice a survey was completed a day before the invitation was sent out (“Rehab: 2nd week” and “Rehab: 3rd week”). * Three outliers were not displayed for readability reasons. The values of these outliers were 26, 39, and 46 days.

Compliance with study procedures was assessed by calculating percentages of weekly survey completion, the proportion of completed surveys per individual (cross-tables in Supplementary Appendix Table AS1), and by the number of days between survey invitation and completion (Figure 1). Sufficient device wear time, defined as at least 10 h of wear time between 6:00 a.m. and 11:00 p.m., was computed and compared before and after rehabilitation stay discharge (Supplementary Appendix Table AS2). Details on the further processing of the PA tracker data in the BarKA-MS study can be found elsewhere (26).

Study participants also provided regular feedback on device experiences in the free text comment fields of the weekly surveys. These free text data consisted mostly of some brief sentences or keywords in German and were examined by use of a word cloud (for the time periods before and after discharge separately). To this end, the free-text entries were manually cleaned and spell-checked. The entries originally written in German were translated into English by DeepL Pro (33). All preprocessing steps were conducted in R, version 4.0.3 (31) using the RStudio environment, version 1.4.1103 (32). The translated texts were assigned parts of speech using the R package “udpipe”, version 0.8.9 (34, 35), subsequently adjectives, nouns, and verbs were extracted, and the remaining words were lemmatized. Key words appearing at least three times in all text entries were visualized as a word cloud using the R package quanteda, version 3.0.0 (36). In addition, the frequency with which each word occurred was examined visually through bar plots created with the R package ggplot2, version 3.3.5 (Supplementary Appendix Figures AS2, AS3).

### 2.5. Qualitative analysis of support logs

Finally, the support logs maintained by the study research team were reviewed, and entries were manually grouped into five scalability challenge domains based on the ISAT checklist according to their content (Supplementary Appendix Table AS5, Part B). In addition, the scalability of each support log observation was qualitatively assessed for potential scalability according to the ISAT scales: no scalability, to a small extent, somewhat, and to a large extent. The grouping and scalability assessment was performed by the first author and reviewed by the last author.

## 3. Results

### 3.1. Recruitment, attrition, and study participants

#### 3.1.1. Recruitment and attrition

**Figure 2:**
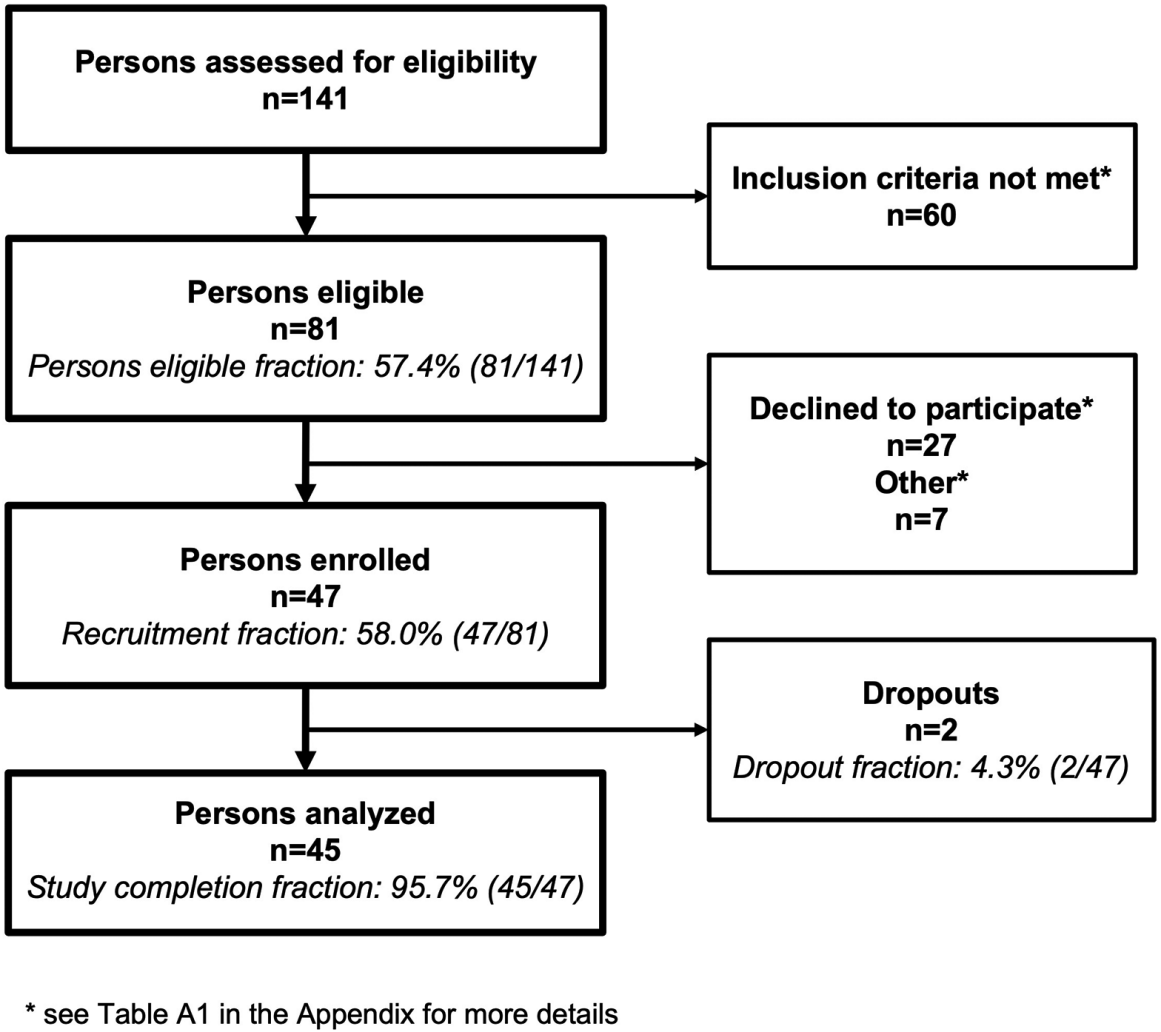
Flow chart of the study population. In total, 141 persons were assessed for eligibility, 47 were enrolled, and the data of 45 persons were analyzed. Unmet inclusion criteria and the reasons for declining study participation are presented in Supplementary Table AS3.

Recruitment occurred between January and September 2021. During that period, 141 PwMS attended the rehabilitation clinic in Valens and were screened for study participation eligibility (Figure 2). Among these persons, 81/141 (57.4%) were eligible, and from these 47/81 (58.0%) wished to participate and were enrolled. Of the persons not meeting the inclusion criteria, 23/60 (38.3%) did not meet the EDSS score requirements (Supplementary Appendix Table AS3). Of the enrollees, 2/47 (4.3%) dropped out for reasons unrelated to the study and disease level. One person, with an EDSS of 2.5, left the rehabilitation program early and the second person with an EDSS of 5, attended a second rehabilitation clinic almost immediately after returning home. In total, 45/47 persons (95.7%) completed the BarKA-MS study and remained in the study for 7 weeks (range 6–8 weeks) on average.

#### 3.1.2. Study participant characteristics

Of the study completers, 29/45 (64.4%) were female and 19/45 male (35.6%) (Table 2; characteristics of non-eligible persons and dropouts are shown in Supplementary Appendix Table AS4). The median age was 46 [interquartile range (IQR) 40–51] years, and 34/45 (75.6%) of the participants had Swiss nationality. All participants were below the retirement age of 64 years for women and 65 years for men in Switzerland. Of the participants, 18/45 (40%) were not working, 17/45 (37.8%) were working 50% or less, 5/45 (11.1%) were working part-time but more than 50%, and 5/45 (11.1%) were working full-time. The majority of participants either had secondary-progressive MS (19/45, 42.2%) or relapsing-remitting MS (18/45, 40%). The median disease duration (measured from diagnosis) was 11 years (IQR 5–21). The EDSS distribution was as follows: 13/45 (28.9%) had an EDSS ≤3.5, 19/45 (42.2%) had an EDSS between 4.0 and 5.5, and 13/45 (28.9%) had an EDSS ≥ 6.0.

**Table 2:** Demographic and health characteristics, as well as baseline assessments of the study participants (*n* = 45).

Characteristics	Study participants (*n* = 45)
Demographic information	
**Sex, *n* (%)**
Female	29 (64.4%)
Male	16 (35.6%)
Age, median (IQR)	46 (40-51)
**Nationality^a^, *n* (%)**	
Swiss	34 (75.6%)
German	6 (13.3%)
Italian	2 (4.4%)
Other	3 (6.7%)
**Marital status, *n* (%)**	
Single	12 (26.7%)
Married	23 (51.1%)
Separated	1 (2.2%)
Divorced	7 (15.6%)
Widowed	2 (4.4%)
**Education, *n* (%)**	
Mandatory school not completed (or up to and including 7th grade)	2 (4.4%)
Apprenticeship or secondary education completed (i.e. Matura schools or intermediate diploma schools)	25 (55.6%)
Higher professional education, applied university or university completed	18 (40%)
**Employment, *n* (%)**
Working full time	5 (11.1%)
Working more than 50% but less than 100%	5 (11.1%)
Working 50% or less	17 (37.8%)
Not working	18 (40%)
**Health information**
**Multiple sclerosis type, *n* (%)**	
Relapsing-remitting multiple sclerosis	18 (40%)
Secondary-progressive multiple sclerosis	19 (42.2%)
Primary-progressive multiple sclerosis	8 (17.8%)
Multiple sclerosis duration, median (IQR)	11 (5-21)
Expanded Disability Status Scale, median (IQR)	4 (3.5-6.0)
**Expanded Disability Status Scale, *n* (%)**	
0–3.5	13 (28.9%)
4–5.5	19 (42.2%)
≥6	13 (28.9%)
Time since last relapse in years, median (IQR)	3 (1-5)
Missing information	8
Body mass index, median (IQR)	24 (21-28)
Missing information	4
**Comorbidities^a^, *n* (%)**
None	18 (40%)
Hypertension	5 (11.1%)
Depression	5 (11.1%)
Skin diseases (e.g., acne)	4 (8.9%)
Orthopedic diseases (e.g., joint or back pain)	4 (8.9%)
Diabetes type II	3 (6.7%)
Migraine	2 (4.4%)
Hypothyroidism	2 (4.4%)
Other^b^	9 (20%)
**Baseline assessments**
** Change in the amount of sport practiced after the MS diagnosis, *n* (%)**	
Less	27 (60%)
Same amount	2 (4.4%)
More	15 (33.3%)
Missing information	1 (2.2%)
Barriers to Health Promoting Activities for Disabled Persons Scale (score range 18-72), median (IQR)	28 (26-32)
International Physical Activity Questionnaire—Short Form, median (IQR) of total active minutes per day in the last seven days	155 (90-240)
Missing information	4
12-Item Multiple Sclerosis Walking Scale (score range 0-100; **refers to the last two weeks**), median (IQR)	62.5 (39.6-81.2)
Missing information	2
**Fatigue Scale for Motor and Cognitive Functions (score range 20-100; refers to the everyday life), *n* (%)**	
No fatigue (score <43)	4 (8.9%)
Mild fatigue (score ≥43)	15 (33.3%)
Moderate fatigue (score ≥53)	3 (6.7%)
Severe fatigue (score ≥63)	23 (51.1%)
** Fatigue Scale for Motor and Cognitive Functions–Cognitive fatigue (score range 10-50; refers to the everyday life), *n* (%)**
No cognitive fatigue (score <22)	18 (40%)
Mild cognitive fatigue (score ≥22)	4 (8.9%)
Moderate cognitive fatigue (score ≥28)	9 (20%)
Severe cognitive fatigue (score ≥34)	14 (31.1%)
** Fatigue Scale for Motor and Cognitive Functions–Motor fatigue (score range 10-50; refers to the everyday life), *n* (%)**
No motor fatigue (score <22)	1 (2.2%)
Mild motor fatigue (score ≥22)	5 (11.1%)
Moderate motor fatigue (score ≥27)	6 (13.3%)
Severe motor fatigue (score ≥32)	33 (73.3%)
General Self-Efficacy Scale (score range 10-40), median (IQR)	30 (28-33)
** Patient Health Questionnaire-8 (score range 0-24; refers to the current state), n (%)**
No significant depressive symptoms (score <5)	19 (42.2%)
Mild depressive symptoms (score ≥5)	17 (37.8%)
Moderate depressive symptoms (score ≥10)	6 (13.3%)
Moderately severe depressive symptoms (score ≥15)	2 (4.4%)
Missing information	1 (2.2%)
EuroQol Visual Analogue Scale (value, 0-100 scale; **refers to “today”**), median (IQR)	60 (50-75)
Missing information	1
EuroQol 5-Dimension 5-Level weighted by the French value set (0-100 scale; **refers to “today”**), median (IQR)	63.5 (46.7-73.0)
Missing information	2
How bad was your pain when it was at its lowest during the last 7 days? (0-10 scale), median (IQR)	0 (0-1)
How bad is your pain right now? (0-10 scale), median (IQR)	1 (0-3.2)
Missing information	5
How bad was your pain when it was at its worst during the last 7 days? (0-10 scale), median (IQR)	3 (0-6)
Walking endurance: 6 Min Walk Test [meter], median (IQR)	329.5 (205-420.5)
Missing information	1
Walking speed: 10 Meter Walk Test [second], median (IQR)	9 (7-13)
Missing information	0
Balance and dynamic functional mobility: Timed Up and Go [second], median (IQR)	10 (8-14)
Number of days in the rehabilitation clinic, median (IQR)	22 (18-26)

^a^ Multiple answers possible.

^b^ Asthma, diabetes type I, osteoporosis, psoriasis, cancer, rheumatic diseases, elevated cholesterol, colitis ulcerosa, fibromyalgia, shingles, Meniere's disease, cerebellar syndrome.

Overall, 27/45 (60%) of the study participants stated they decreased their level of PA and 15/45 (33.3%) stated they increased their level of PA after the MS diagnosis. At study enrollment, participants reported a median of 155 (IQR 90–240) daily active minutes in the last 7 days (including the pre-rehabilitation period) in the International Physical Activity Questionnaire—Short Form questionnaire (encompassing walking, moderate, and intense PA. In total, 26/45 (57.8%) of the participants presented with moderate to severe fatigue.

### 3.2. Adherence to study procedures

#### 3.2.1. Survey completion

Overall, 342/354 (96.6%) of the surveys sent out were completed on time (i.e., latest 2 days before the completion of the next survey). Among the study participants, 35/45 (77.8%) had a completion rate of 100% (Supplementary Appendix Table AS1), while 8/45 (17.8%) missed one survey, and 2/45 (4.4%) missed two surveys. For the latter two participants, the lower compliance was also a consequence of a technical problem in the survey platform hindering the sending of invitations to complete the questionnaires.

Completion of the weekly surveys ranged between 89% and 100% during the rehabilitation phase and between 96% and 100% during the phase back home (Supplementary Appendix Table AS1).

Furthermore, the majority of participants responded promptly to survey invitations, as illustrated by median times of 0 or 1 day elapsed between the invitation and completion of the different surveys in all study phases (Figure 1).

#### 3.2.2. Fitbit wear time

During the rehabilitation stay, on 99% (range: 87% to 100%) of the days, the Fitbit was worn for at least 10 h between 6:00 a.m. and 11:00 p.m., corresponding to a valid wear day (Supplementary Appendix Table AS2). In the home setting, 97% (range: 62% to 100%) of all days were valid wear days (Supplementary Appendix Table AS2). Furthermore, during the rehabilitation stay, 37/45 (82.2%) participants reached 100% valid wear days as compared to 25/45 (55.6%) persons in the home setting phase.

### 3.3. User experiences with devices

**Figure 3:**
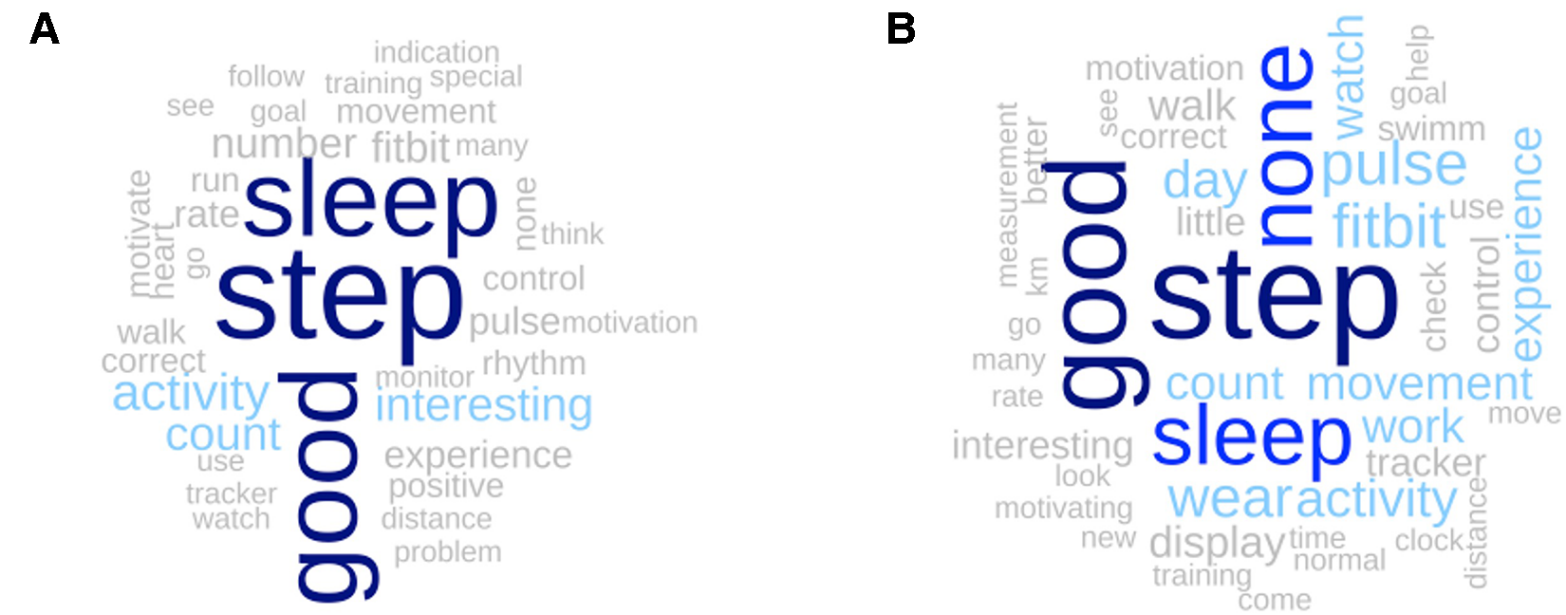
Word cloud of the words appearing at least three times in the weekly answers to the question “what was your experience with activity trackers this week?” asked during the rehabilitation phase (Panel A, n answers = 107) and during the home phase (Panel B, n answers = 142). See Supplementary Figures AS2,AS3 in the Supplementary Appendix for more details about the frequency of the words.

The weekly surveys repeatedly queried study participants about their experience with activity trackers during the past week, both during the inpatient stay and in the home setting. During the rehabilitation phase, 107 answers were captured, which most frequently made references to “step”, “sleep”, and “good” (Figure 3A and Supplementary Appendix Figure AS2), with more than 20 mentions each. The contextual use of these words is illustrated in Table 3 by showing exemplar participant statements that were predominantly positive. In addition, 142 statements were collected during the home phase, with almost identical results. The three most common words were “step”, “good”, and “none”, followed by “sleep” (Figure 3B and Supplementary Appendix Figure AS3). Of note, the word “none” was used to express no new experiences since the inpatient phase. Exemplar statements by study participants are presented in Table 3.

**Table 3:** The three most frequent keywords used by the study participants in the answers given during the rehabilitation phase or the phase back home to the question “what was your experience with activity trackers this week?” together with answer extracts.

Most frequent words	Rank's frequency in rehab	Rank's frequency back home	Study participants’ quotes from the rehabilitation phase	Study participants’ quotes from the phase back home
**Step**	**1**	**1**	“I look at the number of **steps** and walk around some more to reach my goal.” “The tracker sometimes calculates **steps** very generously.” “I walk significantly more **steps** than at the beginning of the study.” “Motivation for the number of **steps**.”	“Helps me reach the goal of 7,000 **steps**.” “Fitbit watch is good to wear. Helpful for counting **steps** and monitoring heart rate while exercising.” “The Fitbit is on average about 30% higher with the **steps** counted than my own smartwatch.” “Motivation to take **steps** has decreased.”
**Sleep**	**2**	**3^a^**	“I find the **sleep** rhythm very interesting.” “Good to see especially the **sleep** cycle.” “I pay attention to **sleep** (duration).” “[The Fitbit] is hooking, especially the **sleep** analysis.”	“**sleep** more controllable.” “I can observe the effect of shorter **sleep**.” “The **sleep**, how much I have REM-phases.” “**Sleep** measurement sometimes inaccurate/incorrect.”
**Good**	**3**	**2**	“Very **good** [experience], I am very satisfied.” “**Very good**. It helps to become aware of what you have done or not done.” “Easy to use and **good** for myself to maintain motivation for achieving the daily goals.” “Still **good**, don’t actually notice the device anymore.”	“**Good** and exciting experience.” “Very **good**! Motivates immensely.” “It went quite **good**, I kept looking in to see how many steps I had walked.” “[I] am not sure if the watch correctly measures my activities otherwise **good** experience.”

REM, rapid eye movement.

^a^ The third most common word was “none”, which was namely used as a finite answer when study participants had no new experience to report, therefore the 4th most common word was used instead.

In total, 42/249 (16.9%) non-empty survey entries had a negative connotation and were referring to problems such as measurement inaccuracies (30 mentions), reduced wear comfort (e.g., during the night or due to skin rash, 6 mentions), and other miscellaneous difficulties such as unintuitive user interface or data loss.

### 3.4. Review of support logs, lessons learned, and scalability

A summary of identified challenges, facilitating factors, and lessons learned from the support logs are presented in Table 4. Additionally, each of the identified points was cross-referenced with the suitable five axes of the ISAT scalability checklist (Supplementary Appendix Table AS5, Part B). In total, we identified 25 such topics, which we classified into “Effectiveness of support measures” (mostly referring to the ISAT axes 1 “fidelity and adaptation” and 4 “implementation infrastructure”), “Recruitment and compliance barriers” (ISAT axes 2 “reach and acceptability” and 3 “delivery setting and workforce”), and “Technical challenges” (ISAT axis 4 “implementation infrastructure”) in Table 4. We performed a qualitative assessment of the challenges encountered and our support for their potential scalability.

**Table 4:** Effectiveness of support measures, recruitment and compliance barriers, technical challenges, and lessons learned in the conduction of the BarKA-MS study and scalability potential.

#	Key words	Description	Lessons learned	Concept reflected in following ISAT axes	Scalability potential Assessment (based on ISAT scale) 1) Not at all 2) To a small extent 3) Somewhat 4) To a large extent
**Effectiveness of support measures**
1	**Comprehensive onboarding**	At the baseline meeting, the on-site study coordinator at the rehabilitation clinic equipped the study participants with a Fitbit, helped them download the Fitbit application onto their mobile phone, and helped with the first log in to their Fitbit study account. Next, the study coordinator created a new account for the study participant on the study platform (RMIS), checked that they could receive emails on their phone, and then completed the baseline questionnaire with them. This initial onboarding session lasted approximately one hour.	It was important to take time to discuss the study in detail with the participants and answer all their questions as the study involved quite a number of digital tools.	ISAT 1: “fidelity and adaptation”	Somewhat
2	**Personal relationship**	The on-site study coordinator had regular contact with the study participants and made sure that everything was going well during the rehabilitation phase. Also, the digital device expert team was always reachable for study participants by phone.	Regular contact with the study participants strengthens their commitment to the study.	ISAT 1: “fidelity and adaptation”	To a small extent
3	**Research team support for on-site study coordinator**	The on-site study coordinator performed the study participant onboarding but initially had limited experience in using digital tools. Despite this, everything worked well due to an initial knowledge transfer by the research team and a smooth hand-over to other experts in the research team for more complex technical questions.	Briefing the on-site study coordinator before the study start and being at their disposal (e.g., per phone) in case of a (technical) issue or questions was essential for the smooth conduction of the study.	ISAT 1: “fidelity and adaptation” and ISAT axis 4: “implementation infrastructure”	To a large extent
4	**Active monitoring**	Research team closely monitored the participants. They controlled that the questionnaires were filled in every week and that the Fitbit were collecting data. If a questionnaire had not been completed or if Fitbit data were not being collected, the research team would pro-actively contact the study participants.	The collection of high-quality and complete data requires active monitoring and is, therefore, resource consuming.	ISAT axis 4: “implementation infrastructure” and ISAT axis 5: “sustainability”	Somewhat
5	**Monitoring device satisfaction and adherence**	Wearable devices, especially the Fitbit Inspire HR, was appreciated by the study participants, who also reported increased motivation to be physically active.	The convenience and discretion of the Fitbit Inspire HR and its immediate feedback were valued by the study participants and seemed to have led to very high wear adherence.	ISAT axis 2: “reach and acceptability”	To a large extent
6	**Offering free Fitbit device as an incentive**	Some people participated in the study mainly because the Fitbit was offered free-of-charge at the end of the study.	Offering a reward to the study participants can increase the recruitment fraction.	ISAT axis 4: “implementation infrastructure”	Somewhat
7	**Involving significant others for peer support**	The study participant's partner was sometimes of great support e.g., for study participants not having German mother tongue.	A study participant's partner can be an additional and valuable support for the participant in terms of study compliance.	Not applicable	To a small extent
**Recruitment and compliance barriers**
8	**Recruitment challenges**	Conducting the study in a rehabilitation clinic led to a limited enrollment fraction (33%) due to the exclusion of persons with very advanced gait impairments.	The PwMS attending a rehabilitation stay tend to have a more advance disease stage. By choosing a different study implementation setting, a higher recruitment fraction could be reached.	ISAT axis 3: “delivery setting and workforce”	To a small extent (limited influence on source population)
9	**COVID-19**	The study took place in 2021, during the COVID-19 pandemic, which led to delay in participant recruitment, as less PwMS attended the clinic for a rehabilitation stay.	The recruitment of PwMS was conditional on PwMS entering a rehabilitation stay. Again, by choosing a different study implementation setting, more PwMS could be reached and thus the recruitment speed could increase.	ISAT axis 3: “delivery setting and workforce”	Not applicable
10	**Seasonality**	During summer, less persons attended the rehabilitation clinic. It was thus more difficult to recruit participants during this period.	In planning the study, seasonality should be taken into consideration e.g., during the main holiday periods (in summer and at Christmas) people are less prone to attend a rehabilitation clinic.	ISAT axis 2: “reach and acceptability”	Somewhat (limited influence on clinical workflows and processes)
11	**Digital literacy**	Persons with less digital literacy required closer support.	When recruiting study participants, make sure that they understand well enough the technologies used and provide easy-to-understand instructions. This could be an inclusion criterion.	ISAT axis 2: “reach and acceptability”	Somewhat (exclusion could lead to limited generalizability)
12	**Language barriers**	Study participants with a different mother tongue than the one in which the study was conducted required more support.	If the person is not fluent in the languages in which the study is performed, this will pose a recruitment obstacle or may lead to a higher participation burden for study enrollees. Additional support (e.g., by relatives or study personnel) could be considered.	ISAT axis 2: “reach and acceptability”	Somewhat
13	**Setting change**	Survey completion fraction at home was lower than in inpatient settings. The participants’ return home was always a key moment because they were not so closely followed anymore, and the daily concerns were again present.	Sending a text message to participants a few days after their return home to remind them that they can reach us anytime if they need to could potentially increase their compliance to the study.	ISAT axis 3: “delivery setting and workforce”	To a large extent
14	**Text messages vs. phone calls**	Study participants would rarely pick up the phone, especially when it is an unknown number, but they would respond to text messages.	The participants in our study (solely PwMS) seemed to feel more at ease in responding to text messages or to calls that were scheduled in advance.	ISAT axis 2: “reach and acceptability”	Somewhat
15	**Forgotten Passwords**	Some study participants lost the password to their RMIS account or went on holiday without it.	The provision of a study “visiting card” with the login information could be provided to the study participants, so that they can easily keep it in their wallet. Participants should be reminded to also take this card on holiday.	ISAT 1: “fidelity and adaptation” and ISAT axis 5: “sustainability”	To a large extent
16	**New mobile phone**	When a study participant changed their phone during the course of the study, they had to re-install the Fitbit application, log in again, and reconnect the application with their Fitbit tracker. Not every study participant would be able to do this on their own.	Manuals and instructions (e.g. short videos) could be provided to advise participants on how to set up a new phone.	ISAT 1: “fidelity and adaptation” and ISAT axis 5: “sustainability”	Somewhat
**Technical challenges**
17	**Incompatible mobile phone operating systems**	Certain mobile phones of the Huawei brand did not have an apps store, which hindered the download of the Fitbit application and thus participation in the study.	Be aware that not all mobile phones have an app store.	ISAT axis 4: “implementation infrastructure”	Not at all (unless phones are provided by study)
18	**No email access on mobile phone**	Some study participants had no access to their emails on their mobile phone. In such a case, the contact person in the rehabilitation clinic had to link the email account of the study participant to an email application on the participant's phone. This was not always successful because e.g., the participant did not have their email account credentials available.	Instruct participants to bring along their login credentials to enable on-site installation and set-up of necessary applications by study supporters.	ISAT 1: “fidelity and adaptation” and ISAT axis 4: “implementation infrastructure”	Somewhat
19	**Provision of a notebook during the rehabilitation stay**	For some participants, it was difficult to complete the weekly questionnaires on their mobile phones, e.g. due to small fonts or hand control impairments.	The provision of a notebook, at least for the initial inpatient phase, should be considered.	ISAT axis 4: “implementation infrastructure”	To a large extent (for inpatient setting)
20	**Login problems with survey platform**	Sometimes during the rehabilitation stay, because of login problem to the RMIS survey platform or other difficulties to fill in the survey digitally, surveys were filled in on paper. However, afterwards the data had to be manually entered by the research team on the RMIS platform, which was time consuming with the added risk of errors in entries.	For different reasons, study participants were not always able to fill in the surveys digitally. A fallback option such as paper questionnaires could be considered, which may lead to a subsequent higher workload and potential data entry errors due to manual entry.	ISAT axis 4: “implementation infrastructure”	To a small extent
21	**Email bounces**	For unclear reasons, the RMIS platform could not send emails to certain email addresses provided by the study participants.	Asking the study participants for an alternate email address or other contact information at the beginning of the study could be useful in different situations.	ISAT axis 4: “implementation infrastructure”	To a large extent
22	**Replies to automatically generated emails**	Participants would sometimes reply to automatically generated emails from the RMIS platform to ask for help or mention a problem, which led to delays in their resolution.	When using automatically generated emails, a note should be added at the end to indicate not to reply to this email address.	ISAT axis 4: “implementation infrastructure”	To a large extent
23	**Automatically timed invitation and reminder emails**	Manually managing email invitations and reminders is labor intensive, with the burden and complexity growing exponentially with each additional participant. The setting transition also needed to be reflected in survey delivery: the first home setting survey differed from normal weekly surveys and had to be triggered manually.	The study platform ideally contains all necessary features for a proper and efficient study conduction, including automated timing of survey and invitation emails, as well as allowing for different study phases.	ISAT axis 4: “implementation infrastructure”	If manual: To a small extent
24	**Extension of the rehabilitation stay in progress**	The rehabilitation stay was sometimes prolonged during the stay by the health insurance. These changes were communicated to Kliniken Valens, who then had to inform the research team of the UZH. This made the weekly survey mailing dates planning challenging.	Rehabilitation stays can be extended and the study platform used should be flexible enough to account for such changes.	ISAT axis 4: “implementation infrastructure”	If manual: To a small extent
25	**Synchronization issue with the Fitbit**	The Fitbit did not always synchronize automatically with participants’ mobile phones. When this happened, the participants had to manually synchronize the Fitbit every few days for the entire study period.	Fitbit devices and synchronization status require regular monitoring (e.g., as available in the Fitabase data platform). If necessary, the study participants should be informed and instructed on how to manually synchronize the Fitbit with the Fitbit application.	ISAT axis 4: “implementation infrastructure”	To a small extent

PwMS, people with MS; RMIS, Research Management Information System.

To broadly summarize, the successful execution of the BarKA-MS study was primarily based on three cornerstones. (1) The availability of a study coordinator on-site at the rehabilitation clinic enabled the building and maintaining of a trusting relationship between study participants and the research team, especially for the home setting phase. Indeed, 22/45 (48.9%) study participants were contacted *via* text message or phone call during the second study phase. The two main causes were the non-completion of a weekly survey after 2 days and the non-synchronization of the Fitbit tracker with the participant's mobile phone. (2) The close collaboration between on-site personnel at the clinic and the outside research team in designing the study led to an optimized workload distribution (according to individual strengths) and enabled an efficient collaboration between on-site study coordinators and the research team. (3) The use of well-accepted Fitbit devices, along with the onboarding procedures, pro-active remote monitoring, and remote support enabled participants to overcome technical challenges and enabled a positive experience with the Fitbit devices.

We also encountered some challenges along the way: (1) study recruitment was impaired by the COVID-19 pandemic and the summer holidays, thus requiring a longer overall recruitment period than initially envisioned. (2) Getting in contact with study participants posed some challenges as they rarely answered phone calls from an unknown number and multiple contact attempts were often needed, and (3) the remote study support turned out to be quite time-consuming due to a multitude of Fitbit usability challenges, including participants forgetting their password, needing support in restoring the app and device connection, as well as user errors.

## 4. Discussion

This analysis presents a recently conducted mHealth study of 45 PwMS who wore a consumer-grade fitness tracker device during 6–8 weeks —the BarKA-MS study. Our analysis critically examined study recruitment and participant compliance with study procedures, user experiences with the wearable devices, as well as the scalability of such an mHealth study.

In the BarKA-MS study, we attained an overall high recruitment fraction of about 58% (*n* = 47) among 81 eligible participants, and only two dropouts were registered. Also, we achieved a high completion of weekly surveys and a high daily fitness tracker wear time of over 90%. Additionally, study participants expressed a strong enthusiasm toward Fitbit use in the beginning and reported an increased motivation to be physically active. Last, we identified and cross-referenced 25 topics with the five axes of the ISAT checklist. A thorough onboarding, the creation of a trusting relationship, and participant support were important factors for study compliance, but support scalability is limited.

The inclusion criteria of the BarKA-MS study led to the *a priori* exclusion of a relatively large fraction of initially screened PwMS. In this regard, not matching the required EDSS range was the most restraining factor of the recruitment. That is, many persons attending the rehabilitation clinic were using wheelchairs and were therefore excluded due to the BarKA-MS study focus on daily step counts and PA. Among eligible persons, our recruitment fraction was about 10% higher than those reported in the literature (37).

Furthermore, in regard to study guidelines adherence and data quality, our study exposed comparatively high study compliance and retention. This contrasts with other reports of substantial study compliance issues in remote digital health studies already a few weeks into the study follow-up (38–40). In the BarKA-MS study, compliance was likely enhanced by the two-phase design of our study with onsite recruitment and onboarding, complemented by low-level remote support and pro-active monitoring for technical issues with devices and the study platform. Similar measures were also found to be effective by other studies (41).

In addition, several factors affected not only recruitment and onboarding, but also adherence and data quality. Our findings highlight the substantial demands on digital and health literacy for digital health study participation. Specifically, participants needed to be in possession of a compatible smartphone and have at least some basic digital literacy skills (e.g., for installing and utilizing apps). Indeed, several studies referred to the lack of knowledge about digital technologies by study participants and study personnel alike (9, 42, 43), and tool complexity (44, 45) as substantial barriers to technology adoption. One of these studies also made positive experiences with onboarding sessions for study participants and the availability of coaches and/or a support system for facilitating study participation (42). Our experiences further showed that language skills could pose an obstacle to recruitment and study task execution (with the surveys only being available in German). Study inclusion was further restricted by requiring the ability of self-ambulation, thus excluding PwMS who use a wheelchair at home and have a more advanced disease state. Compared with the national Swiss Multiple Sclerosis Registry, the population in the BarKA-MS study tended to be somewhat younger, but the proportions of primary and secondary progressive MS disease stages were even higher (46).

We further performed qualitative evaluations of the user experiences and feedbacks relating to Fitbit device satisfaction. In general, the qualitative assessment of survey-collected user experiences suggests the devices were well-liked and accepted, thus underscoring their potential for longer-term observations. Furthermore, several persons reported how the monitoring of steps, PA, or sleep provided motivation and enabled self-observations, which was also seen in other studies (12, 47, 48). Nevertheless, as the initial enthusiasm waned, some negative points became more apparent. Specifically, participants remarked inaccuracies in sleep assessments and step counts. Rarely, participants also mentioned technical issues, non-intuitive user interfaces, and wear discomfort during regular follow-up surveys and support calls.

Finally, we reviewed the support logs and summarized the technical issues and barriers, but also positive experiences with implemented support measures. Our review highlights two major aspects. A key finding from this review was that a well-designed, comprehensive onboarding and participant support system can contribute to greater study compliance, which was also noted by other studies (41). In the BarKA-MS study, especially the individualized onboarding sessions (#1, Table 4), the face-to-face contacts during the rehabilitation inpatient stay (#2), and close monitoring and individualized technical support (#3 and #4) were well received by participants. Other studies also found that compliance is likely associated with the number and duration of direct participant interactions (49). However, due to the essential involvement of the on-site study coordinator and the research team for remote support in the BarKA-MS study, these measures are not easily scalable. For example, technical training and onboarding at the beginning of recruitment can be streamlined to some extent by providing adequate training material. However, many of the support requests during the study required highly individualized problem solutions and time-consuming follow-ups.

The ISAT tool provided useful guidance for structuring and evaluating our study design with respect to future scalability. However, having primarily been developed for non-digital interventions, the ISAT tool does not entirely cover all scalability challenges identified by our study. For example, prospective users need adequate digital and health literacy skills (50), as well as the financial means to buy the devices (24). Such skills were not limited to the ability to use and manage electronic devices but also included understanding and processing information and general health literacy to be able to follow study instructions (50). These and other accessibility hurdles were addressed in the BarKA-MS study by supportive actions to help prospective study participants in setting up and using the devices. Furthermore, adherence is likely to be associated with the number or duration of contact of the participants with the study coordinators (49). In the BarKA-MS study, participants had regular face-to-face contact and received ongoing support during the rehabilitation stay. Therefore, scalability is not only an issue of increasing study participant numbers but also of the duration of studies. Overall, we found that these issues are currently not well reflected by the ISAT checklist, and further, mHealth-specific adaptations may be warranted.

Combined, our findings suggest that digital health researchers may be confronted with a compliance-scalability trade-off. While direct and individualized interactions between study conductors and participants contribute to trust-building, enhanced participant commitment, and better study task completion, the strong human involvement makes the provision of such support elements potentially very costly as the number of study participants increases. Unfortunately, there is currently no easy solution to overcome this trade-off. Possible strategies may include optimizing the support level, human interactions and, in parallel, increasing the number of study participants to compensate for the likely greater attrition loss (51). Additionally, technological advances such as health diaries with integrated reminders (8, 52), chatbots or conversational agents (53, 54), and *Just-In-Time Adaptive Intervention,* which enables support when users are in a receptive state (55–57), could potentially be leveraged to provide scalable user support. Intervention adherence can also be enhanced through remote support (e.g., text messaging, emails, video calls) (37, 58), and remote program participation, such as web-based physiotherapy (8, 58, 59). Remote program participation offers greater flexibility in terms of participation time (8, 58, 59). But ultimately, the economical and efficiency aspects of developing and operating remote digital health studies are clearly under-researched and warrant greater attention.

Some limitations of the present analysis and the BarKA-MS study in general should be ­noted. First, the BarKA-MS study has a limited sample size and included only up to 8 weeks of follow-up. We were therefore unable to derive conclusions about longer-term barriers and challenges. Also, the included sample does not reflect the full diversity of PwMS with respect to age, disability status, or digital skills. Furthermore, the support of the on-site coordinator during the completion of the baseline survey may have led to information biases, especially in the well-being-related questionnaires (i.e., physical activity level, barriers to physical activity, depression, walking ability, fatigue, health-related quality of life, pain, and self-efficacy). However, as these data were not analyzed here, this has a limited impact. Moreover, the support from the on-site coordinator was a chance to bind with the study participant and build a relationship, which likely had a positive effect on compliance (60, 61). Despite our efforts to review and qualify our data by two separate reviewers, the analyses and conclusions presented here are ultimately qualitative and, to some extent, subjective. Our findings should be considered formative and interpreted with appropriate caution. Furthermore, although the ISAT tool provided a helpful framework for our scalability assessments, it was not specifically designed for mHealth studies and recently has been qualified to require further validation by a systematic review (25). Therefore, relevant scalability elements or axes could have been missed by our analysis. Nevertheless, our detailed methodological critique of the BarKA-MS study design may provide inspiration and potential guidance for other researchers planning similar study efforts.

In conclusion, the BarKA-MS study shows that consumer-grade fitness trackers can be a useful alternative to research-grade devices in digital health studies. The mostly positive user feedback and high wear time observed in our study point to high satisfaction among the study participants. Our experiences also clearly emphasize the importance of an adequate onboarding and participant support system to maintain compliance. Overall, these findings suggest that, in principle, longer-term, remote observations beyond 8 weeks (as in the BarKA-MS study) may be feasible. However, given fixed resources, an increase in sample size may require reducing the level of human-dependent study participant support, with likely consequences for study compliance. Study conductors should anticipate this potential compliance-scalability trade-off already in the design phase.

## Data Availability

The raw data supporting the conclusions of this article will be made available by the authors, without undue reservation.

## References

[1] WHO Global Observatory for eHealth. Mhealth: New horizons for health through mobile technologies: Second global survey on eHealth. Geneva: World Health Organization (2011).

[2] Sim I. Mobile devices and health. N Engl J Med. (2019) 381(10):956–68. 10.1056/NEJMra1806949 31483966

[3] Fatehi F Samadbeik M Kazemi A. What is digital health? Review of definitions. Stud Health Technol Inform. (2020) 275:67–71. 10.3233/SHTI200696 33227742

[4] Steinhubl SR Muse ED Topol EJ. The emerging field of mobile health. Sci Transl Med. (2015) 7(283):283–6. 10.1126/scitranslmed.aaa3487 PMC4748838 25877894

[5] Matthews PM Block VJ Leocani L. E-health and multiple sclerosis. Curr Opin Neurol. (2020) 33:271–6. 10.1097/WCO.0000000000000823 32324706

[6] Bradshaw MJ Farrow S Motl RW Chitnis T. Wearable biosensors to monitor disability in multiple sclerosis. Neurol Clin Pract. (2017) 7(4):354–62. 10.1212/CPJ.0000000000000382 29185551 PMC5648206

[7] Cohen JA Trojano M Mowry EM Uitdehaag BMJ Reingold SC Marrie RA. Leveraging real-world data to investigate multiple sclerosis disease behavior, prognosis, and treatment. Mult Scler J. (2020) 26(1):23–37. 10.1177/1352458519892555 PMC6950891 31778094

[8] Gromisch ES Turner AP Haselkorn JK Lo AC Agresta T. Mobile health (mHealth) usage, barriers, and technological considerations in persons with multiple sclerosis: a literature review. JAMIA Open. (2021) 4(3):1–10. 10.1093/jamiaopen/ooaa067 PMC8423420 34514349

[9] Scholz M Haase R Schriefer D Voigt I Ziemssen T. Electronic health interventions in the case of multiple sclerosis: from theory to practice. Brain Sci. (2021) 11(2):1–13. 10.3390/brainsci11020180 PMC7913051 33540640

[10] Weidemann ML Trentzsch K Torp C Ziemssen T. Enhancing monitoring of disease progression—remote sensoring in multiple sclerosis. Nervenarzt. (2019) 90(12):1239–44. 10.1007/s00115-019-00817-8 31641794

[11] Frechette ML Meyer BM Tulipani LJ Gurchiek RD McGinnis RS Sosnoff JJ. Next steps in wearable technology and community ambulation in multiple sclerosis. Curr Neurol Neurosci Rep. (2019) 19(10):80. 10.1007/s11910-019-0997-9 31485896

[12] Wendrich K van Oirschot P Martens MB Heerings M Jongen PJ Krabbenborg L. Toward digital self-monitoring of multiple sclerosis: investigating first experiences, needs, and wishes of people with MS. Int J MS Care. (2019) 21(6):282–91. 10.7224/1537-2073.2018-083 31889935 PMC6928580

[13] Chitnis T Glanz BI Gonzalez C Healy BC Saraceno TJ Sattarnezhad N Quantifying neurologic disease using biosensor measurements in-clinic and in free-living settings in multiple sclerosis. npj Digit Med. (2019) 2(1):1–8. 10.1038/s41746-019-0197-7 31840094 PMC6906296

[14] Motl RW Sandroff BM Kwakkel G Dalgas U Feinstein A Heesen C Exercise in patients with multiple sclerosis. Lancet Neurol. (2017) 16(10):848–56. 10.1016/S1474-4422(17)30281-8 28920890

[15] Razazian N Kazeminia M Moayedi H Daneshkhah A Shohaimi S Mohammadi M The impact of physical exercise on the fatigue symptoms in patients with multiple sclerosis: a systematic review and meta-analysis. BMC Neurol. (2020) 20(1):1–11. 10.1186/s12883-020-01654-y 32169035 PMC7068865

[16] Midaglia L Mulero P Montalban X Graves J Hauser SL Julian L Adherence and satisfaction of smartphone- and smartwatch-based remote active testing and passive monitoring in people with multiple sclerosis: nonrandomized interventional feasibility study. J Med Internet Res. (2019) 21(8):e14863. 10.2196/14863 31471961 PMC6743265

[17] Block VJ Bove R Zhao C Garcha P Graves J Romeo AR Association of continuous assessment of step count by remote monitoring with disability progression among adults with multiple sclerosis. JAMA Netw Open. (2019) 2(3):e190570. 10.1001/jamanetworkopen.2019.0570 30874777 PMC6484622

[18] Liu S Han J Puyal EL Kontaxis S Sun S Locatelli P Fitbeat: cOVID-19 estimation based on wristband heart rate using a contrastive convolutional auto-encoder. Pattern Recognit. (2022) 123:108403. 10.1016/j.patcog.2021.108403 34720200 PMC8547790

[19] Block VJ Lizée A Crabtree-Hartman E Bevan CJ Graves JS Bove R Continuous daily assessment of multiple sclerosis disability using remote step count monitoring. J Neurol. (2017) 264(2):316–26. 10.1007/s00415-016-8334-6 27896433 PMC5292081

[20] Dalla-Costa G Radaelli M Maida S Sangalli F Colombo B Moiola L Smart watch, smarter EDSS: improving disability assessment in multiple sclerosis clinical practice. J Neurol Sci. (2017) 383:166–8. 10.1016/j.jns.2017.10.043 29246607

[21] Abbadessa G Lavorgna L Miele G Mignone A Signoriello E Lus G Assessment of multiple sclerosis disability progression using a wearable biosensor: a pilot study. J Clin Med. (2021) 10(6):1–8. 10.3390/jcm10061160 PMC8001885 33802029

[22] Block VJ Gopal A Rowles W Yueh C Gelfand JM Bove R. CoachMS, an innovative closed-loop, interdisciplinary platform to monitor and proactively treat MS symptoms: a pilot study. Mult Scler J - Exp Transl Clin. (2021) 7(1):1–13. 10.1177/2055217321988937 PMC7970691 33796329

[23] WHO. Nine steps for developing a scaling-up strategy. (2010);44.

[24] Milat A Lee K Conte K Grunseit A Wolfenden L Van Nassau F Intervention scalability assessment tool: a decision support tool for health policy makers and implementers. Heal Res Policy Syst. (2020) 18(1):1–17. 10.1186/s12961-019-0494-2 PMC6942323 31900230

[25] Ben Charif A Zomahoun HTV Gogovor A Abdoulaye Samri M Massougbodji J Wolfenden L Tools for assessing the scalability of innovations in health: a systematic review. Health Research Policy and Systems. (2022) 20:1–20. 10.1186/s12961-022-00830-5 35331260 PMC8943495

[26] Polhemus A, Sieber C, Haag C, Sylvester R, Kool J, Gonzenbach R, et al. Non-equivalent, but still valid: Establishing the construct validity of a consumer fitness tracker in persons with multiple sclerosis. *PLOS Digit Heal*. (2023) 2(1):e0000171. 36812638 10.1371/journal.pdig.0000171 PMC9931345

[27] Nittas V Mütsch M Frey T Braun J Puhan MA. Effectiveness of a tailored web app on sun protection intentions and its implications for skin cancer prevention: a randomized controlled trial. PLoS Digit Health. (2022) 1(5):e0000032. 10.1371/journal.pdig.0000032 36812525 PMC9931317

[28] Casey B Coote S Donnelly A. Objective physical activity measurement in people with multiple sclerosis: a review of the literature. Disabil Rehabil Assist Technol. (2018) 13(2):124–31. 10.1080/17483107.2017.1297859 28285547

[29] Sasaki JE Sandroff B Bamman M Motl RW. Motion sensors in multiple sclerosis: narrative review and update of applications. Expert Rev Med Devices. (2017) 14:891–900. 10.1080/17434440.2017.1386550 28956457 PMC6291837

[30] Polhemus AM Haag C Sieber C Sylvester R Kool J Gozenbach R Methodological heterogeneity induces bias on physical activity metrics derived from the actigraph GT3X in multiple sclerosis. Front Rehabil Sci. (2022) 3:1–20. 10.3389/fresc.2022.989658 36518351 PMC9742246

[31] Core Team R. R: A language and environment for statistical computing. Vienna, Austria: R Foundation for Statistical Computing (2020).

[32] RStudio Team. RStudio: integrated development for R. Boston, MA: RStudio, Inc. (2021).

[33] DeepL Pro. Translate text, word docs & other docs securely. [cited 2021 Nov 3]. Available from: https://www.deepl.com/pro?cta=menu-pro/

[34] Straka M Straková J. Tokenizing, POS tagging, lemmatizing and parsing UD 2.0 with UDPipe. CoNLL 2017 - SIGNLL conference on computational natural language learning, proceedings of the CoNLL 2017 shared task: multilingual parsing from raw text to universal dependencies (2017). p. 88–99

[35] Wijffels J. udpipe: Tokenization, Parts of Speech Tagging, Lemmatization and Dependency Parsing with the “UDPipe” “NLP” Toolkit. (2022).

[36] Benoit K Watanabe K Wang H Nulty P Obeng A Müller S. quanteda: an R package for the quantitative analysis of textual data. J Open Source Softw. (2018) 3(30):774. 10.21105/joss.00774

[37] Arafah AM Bouchard V Mayo NE. Enrolling and keeping participants in multiple sclerosis self-management interventions: a systematic review and meta-analysis. Clin Rehabil. (2017) 31(6):809–23. 10.1177/0269215516658338 27401492

[38] Yeroushalmi S Maloni H Costello K Wallin MT. Telemedicine and multiple sclerosis: a comprehensive literature review. J Telemed Telecare. (2020) 26(7–8):400–13. 10.1177/1357633X19840097 31042118

[39] Tallner A Pfeifer K Mäurer M. Web-based interventions in multiple sclerosis: the potential of tele-rehabilitation. Ther Adv Neurol Disord. (2016) 9(4):327. 10.1177/1756285616640684 27366240 PMC4916521

[40] Pratap A Neto EC Snyder P Stepnowsky C Elhadad N Grant D Indicators of retention in remote digital health studies: a cross-study evaluation of 100,000 participants. npj Digit Med. (2020) 3(1):1–10. 10.1038/s41746-020-0224-8 32128451 PMC7026051

[41] Balbim GM Marques IG Marquez DX Patel D Sharp LK Kitsiou S Using fitbit as an mHealth intervention tool to promote physical activity: potential challenges and solutions. JMIR Mhealth Uhealth. (2021) 9:25289. 10.2196/25289 PMC7961407 33646135

[42] Lennon MR Bouamrane MM Devlin AM O’Connor S O’Donnell C Chetty U Readiness for delivering digital health at scale: lessons from a longitudinal qualitative evaluation of a national digital health innovation program in the United Kingdom. J Med Internet Res. (2017) 19(2):e42. 10.2196/jmir.6900 28209558 PMC5334516

[43] Simblett S Greer B Matcham F Curtis H Polhemus A Ferrão J Barriers to and facilitators of engagement with remote measurement technology for managing health: systematic review and content analysis of findings. J Med Internet Res. (2018) 20(7):e10480. 10.2196/10480 30001997 PMC6062692

[44] Giunti G Kool J Rivera Romero O Dorronzoro Zubiete E. Exploring the specific needs of persons with multiple sclerosis for mhealth solutions for physical activity: mixed-methods study. JMIR mHealth uHealth. (2018) 6(2):e8996. 10.2196/mhealth.8996 PMC5889817 29426814

[45] Zhao Y Ni Q Zhou R. What factors influence the mobile health service adoption? A meta-analysis and the moderating role of age. Int J Inf Manage. (2018) 43:342–50. 10.1016/j.ijinfomgt.2017.08.006

[46] Kaufmann M Puhan MA Kuhle J Yaldizli Ö Magnusson T Kamm CP A framework for estimating the burden of chronic diseases: design and application in the context of multiple sclerosis. Front Neurol. (2019) 10:953. 10.3389/fneur.2019.00953 31555205 PMC6742909

[47] Ayobi A Marshall P Cox AL Chen Y. Quantifying the body and caring for the mind: self-tracking in multiple sclerosis. Conference on human factors in computing systems – proceedings. New York, NY, United States: ACM (2017). p. 6889–901.

[48] Gonçalves A-C Leckie T Hunter A Fitzpatrick D Richardson A Hardy B Technology supported rehabilitation for patients of critical illness caused by COVID-19: a protocol for a mixed-methods feasibility study. Int J Ther Rehabil. (2020) 27(10):1–9. 10.12968/ijtr.2020.0102

[49] Bevens W Weiland T Gray K Jelinek G Neate S Simpson-Yap S. Attrition within digital health interventions for people with multiple sclerosis: systematic review and meta-analysis. J Med Internet Res. (2022) 24(2):e27735, 10.2196/27735 35138262 PMC8867299

[50] Scheerder A van Deursen A van Dijk J. Determinants of internet skills, uses and outcomes. A systematic review of the second- and third-level digital divide. Telemat Informatics. (2017) 34(8):1607–24. 10.1016/j.tele.2017.07.007

[51] Daniore P Nittas V von Wyl V. Enrollment and retention of participants in remote digital health studies: scoping review and framework proposal. J Med Internet Res. (2022) 24(9):e39910. 10.2196/39910 36083626 PMC9508669

[52] Zettl UK Bauer-Steinhusen U Glaser T Czekalla J Hechenbichler K Limmroth V Adherence to long-term interferon beta-1b injection therapy in patients with multiple sclerosis using an electronic diary. Adv Ther. (2016) 33(5):834–47. 10.1007/s12325-016-0325-6 27090116

[53] Funk B Sadeh-Sharvit S Fitzsimmons-Craft EE Trockel MT Monterubio GE Goel NJ A framework for applying natural language processing in digital health interventions. J Med Internet Res. (2020) 22(2):e13855. 10.2196/13855 PMC7059510 32130118

[54] Car LT Dhinagaran DA Kyaw BM Kowatsch T Joty S Theng YL Conversational agents in health care: scoping review and conceptual analysis. J Med Internet Res. (2020) 22(8):e17158. 10.2196/17158 PMC7442948 32763886

[55] Mishra V Künzler F Kramer JN Fleisch E Kowatsch T Kotz D. Detecting receptivity for mHealth interventions in the natural environment. Proc ACM interactive, mobile. Wearable Ubiquitous Technol. (2021) 5(2):74. 10.1145/3463492 PMC8680205 34926979

[56] Nahum-Shani I Hekler EB Spruijt-Metz D. Building health behavior models to guide the development of just-in-time adaptive interventions: a pragmatic framework. Health Psychol. (2015) 34(0):1209. 10.1037/hea0000306 PMC4732268 26651462

[57] Nahum-Shani I Smith SN Spring BJ Collins LM Witkiewitz K Tewari A Just-in-time adaptive interventions (JITAIs) in mobile health: key components and design principles for ongoing health behavior support. Ann Behav Med. (2018) 52(6):446–62. 10.1007/s12160-016-9830-8 27663578 PMC5364076

[58] Paul L Renfrew L Freeman J Murray H Weller B Mattison P Web-based physiotherapy for people affected by multiple sclerosis: a single blind, randomized controlled feasibility study. Clin Rehabil. (2019) 33(3):473–84. 10.1177/0269215518817080 30514108

[59] Paul L Coulter EH Miller L McFadyen A Dorfman J Mattison PGG. Web-based physiotherapy for people moderately affected with multiple sclerosis; quantitative and qualitative data from a randomized, controlled pilot study. Clin Rehabil. (2014) 28(9):924–35. 10.1177/0269215514527995 24691218

[60] Garcia A Balasubramanian V Lee J Gardner R Gummidipundi S Hung G Lessons learned in the apple heart study and implications for the data management of future digital clinical trials. J Biopharm Stat. (2022) 32(3):496–510. 10.1080/10543406.2022.2080698 35695137 PMC9378511

[61] Jakob R Harperink S Rudolf AM Fleisch E Haug S Mair JL Factors influencing adherence to mHealth apps for prevention or management of noncommunicable diseases: systematic review. J Med Internet Res. (2022) 24(5):e35371. 10.2196/35371 35612886 PMC9178451

