# Supplementary material for: Feasibility and scalability of a fitness tracker study: Results from a longitudinal analysis of persons with multiple sclerosis
Source: Front Digit Health. 2023 Feb 28;5:1006932. doi: 10.3389/fdgth.2023.1006932 (PMC10012422; doi:10.3389/fdgth.2023.1006932)
Supplement: Supplementary file 1 [file Datasheet1.docx]

# Methods Appendix

##
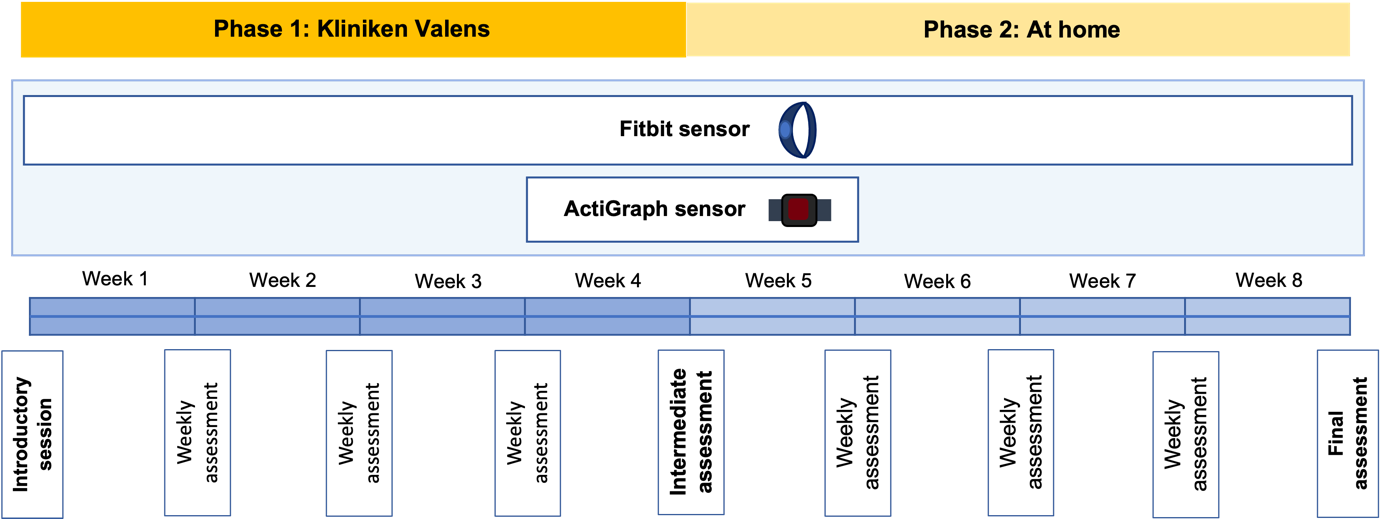
The BarKA-MS Study

**Figure A1.** Timeline of study roll-out with its different phases and assessments.

## Sample Size Determination for the BarKA-MS Study

The BarKA-MS study was intended to provide methodological direction and hypothesis generation for future work. Previous early-stage validation studies which conducted similar analyses to those described here suggest a sample size of 30 to 45 participants to be appropriate (1–3). Further, a central outcome of the present study was the average daily PA. Daily PA assessments in the BarKA-MS study had a two-level hierarchical structure such that PA measurements (level 1) were nested among individuals (level 2). A primary analysis model will examine changes in daily PA over the course of the study (level 1 effect of time). In line with recent recommendations for two-level models (4), a sample size of 30 to 45 participants on level 2 thus allows to detect medium to large effect sizes for the direct effect of time with power ≥ 80 %.

## Instruments

### Research-grade Device

A research-grade device was used in this study, the Actigraph GT3X (Manufacturing Technology, Inc., FL, USA), a three-dimensional accelerometer validated in PwMS (5,6), and initialized in Actilife 6.0 with a sampling rate of 30Hz. Step count, sedentary behavior, and PA duration and intensity can be derived from Actigraph three-dimensional measurements.

Study participants were instructed to wear the Actigraph on their right hip during their last week in Kliniken Valens and their first week back home during waking hours and for a minimum of ten hours.

### Self-reported Measures

**Demographic data** (age, gender, living situation, etc.) and **health status information** (disease type and severity, time since diagnosis, relapse history, comorbidities) were collected in a survey based on a questionnaire developed by the Swiss MS Registry (7) called Layer 2.

Expanded Disability Status Scale (EDSS) (8) categories were defined accordingly to prior studies as mild (EDSS < 4.0), moderate (EDSS 4.0 – 5.5), and severe (EDSS 6.0 – 6.5) (9).

The **Barriers to Health Promoting Activities for Disabled Persons Scale** (BHADP) is an 18-item, 4-point scale (1 = never, 4 = routinely; score range: 18-72) to estimate individual barriers to PA among disabled people, including people with MS (10–12), with no specified time component. The higher the score, the higher the amount of perceived barriers. We translated the BHADP scale from English to German and back to English to ensure the equivalence of both versions.

The **International Physical Activity Questionnaire - Short Form** (IPAQ-SF) is a 7-item self-administered questionnaire to appraise the frequency and duration of PA (vigorous, moderate, walking), as well as time spent sitting per day in the last seven days (13–15).

The **Twelve-Item MS Walking Scale** (MSWS-12) is a 12-item, 5-point scale (1 = „not at all“, 5 = „extremely“; score range: 12-60, transformed into a scale with a range of 0-100) assessing walking ability in the last two weeks among people with MS (16,17).

We used two 11-point visual analog scales (VAS) (0 = „no pain“, 10 = „the strongest pain I can imagine“; score range: 0-10) to assess **pain** at its lowest in the past seven days and pain right now.

The **Fatigue Scale for Motor and Cognitive Functions** (FSMC) is a 20-item, 5-point scale (1 = „absolutely disagree“, 5 = „absolutely agree“; score range: 20-100) to estimate fatigue in everyday life (18). It is composed of two sub-scales, motor and cognitive fatigue, of 10-items each. The score of each of these three scales is categorized into „no [motor/cognitive] fatigue“, „mild [motor/cognitive] fatigue“, „moderate [motor/cognitive] fatigue“, and „severe [motor/cognitive] fatigue“ (see Table 2 for the cut-off values).

**General Self-Efficacy Scale** (GSE) is a 10-item, 4-point scale (1 = „not at all true“, 4 = „exactly true“, score range: 10-40) originally developed in German to assess one’s beliefs in one’s ability to succeed in a particular task or situation with no specified time component (19,20).

The **Eight-item Patient Health Questionnaire depression scale** (PHQ-8) is an 8-item, 4-point scale (0 = „not at all“, 3 = „nearly every day“; score range: 0-24) evaluating depression status in the last two weeks (21). Its score was classified into: „no significant depressive symptoms (score < 5)“, „mild depressive symptoms (score ≥ 5)“, „moderate depressive symptoms (score ≥ 10)“, „moderately severe depressive symptoms (score ≥ 15)“, and „severe depressive symptoms (score ≥ 20)“ (21,22). A score of ≥ 10 defines current depression (22).

The **EuroQol-5 Dimension** (EQ-5D) is a non-disease-specific instrument to assess health-related quality of life with a time component being „today“ (23). The first section, the **five-level EuroQol-5 Dimension** (EQ-5D-5L), is based on five dimensions: mobility, ﻿self-care, usual activities, pain/discomfort, and anxiety/depression; each dimension is further divided into five levels of ability/wellbeing. The French value set was used to weight the EQ-5D-5L (24–26). In the second section, users can estimate their well-being on a VAS ranging from 0 to 100, and by writing down a number between 0 and 100. In both sections, zero represented the lowest health.

The **weekly diary** entailed five questions about study participants’ day-to-day experiences with PA and tracker devices: (1) What were your PA goals this week? (2) What kept you from being physically active this week? (3) What made it easier for you to be physically active this week? (4) What would have been helpful to be more physically active? (5) What's your experience with activity trackers this week?

### Physical Capacity Assessments

The **6-minute Walk test (6MWT)** is a measure of exercise tolerance developed for people with disabilities (27,28). Walking aids were allowed. The distance walked in 6 minutes gives an indication of physical health (27,28). This test was validated among people with multiple sclerosis (PwMS) (29). The study participants were instructed to walk at a comfortable and safe speed.

The **10 Meter Walk Test (10mWT)** is a test to measure walking speed over 10 meters. Walking aids were allowed. The speed gives an indication of walking quality (30). This test exhibited good interrater reliability among PwMS (31).

During the **Timed Up and Go (TUG)** the person, sitting on a chair at the beginning of the test, must stand up, walk 3 meters, turn, walk back, and sit again (32). Walking aids were allowed. The time required to accomplish this mirrors the balance and dynamic functional mobility of the person (32,33). This test displayed strong validity among PwMS (33).

# Results Appendix

## ­Data Completeness

### Completion of the Surveys

**Table A1.** Completion of the weekly questionnaires on an individual and weekly level for the whole study period. Meaning of the colors used in the table: green, the survey was completed; red, the survey was not completed and labeled as missing; light blue, the survey was not sent out for completion and was thus not labeled as missing. The latter case happened when a participant would not be spending 4 weeks in rehabilitation.

| **IDs** | **EDSS** | **Baseline** | **Reha - week 1** | | **Reha - week 2** | | **Reha - week 3** | | **Reha - week 4** | | **End of Rehabilitation** | | **At home - week 1** | | **At home - week 2** | | **At home - week 3** | **Follow-up** | | **# of surveys filled out** | | **# of surveys sent** | **% of surveys filled out** | |
| --- | --- | --- | --- | --- | --- | --- | --- | --- | --- | --- | --- | --- | --- | --- | --- | --- | --- | --- | --- | --- | --- | --- | --- | --- |
| **1** | 6 | V | NA | | X | | X | | X | | V | | V | | NA | | V | V | | 5 | | 7 | 67 | |
| **2** | 3.5 | V | V | | V | | X | | X | | V | | V | | V | | V | V | | 8 | | 8 | 100 | |
| **3** | 4.5 | V | NA | | V | | V | | NA | | V | | V | | V | | V | V | | 8 | | 10 | 78 | |
| **4** | 5.5 | V | V | | X | | X | | X | | V | | V | | V | | V | V | | 7 | | 7 | 100 | |
| **5** | 6 | V | V | | X | | X | | X | | V | | NA | | V | | V | V | | 6 | | 7 | 83 | |
| **6** | 5.5 | V | V | | V | | NA | | X | | V | | V | | V | | V | V | | 8 | | 9 | 88 | |
| **7** | 4 | V | V | | V | | V | | X | | V | | V | | V | | V | V | | 9 | | 9 | 100 | |
| **8** | 4 | V | V | | X | | X | | X | | V | | V | | V | | V | V | | 7 | | 7 | 100 | |
| **9** | 3.5 | V | V | | X | | X | | X | | V | | V | | V | | V | V | | 7 | | 7 | 100 | |
| **10** | 4.5 | V | V | | X | | X | | X | | V | | V | | V | | V | V | | 7 | | 7 | 100 | |
| **11** | 6 | V | V | | V | | X | | X | | V | | V | | V | | V | V | | 8 | | 8 | 100 | |
| **12** | 3.5 | V | V | | V | | V | | X | | V | | V | | V | | V | V | | 9 | | 9 | 100 | |
| **13** | 4 | V | V | | X | | X | | X | | V | | V | | V | | V | V | | 7 | | 7 | 100 | |
| **14** | 6.5 | V | V | | X | | X | | X | | V | | V | | V | | V | V | | 7 | | 7 | 100 | |
| **15** | 6 | V | V | | V | | X | | X | | V | | V | | V | | V | V | | 8 | | 8 | 100 | |
| **16** | 6 | V | V | | V | | X | | X | | V | | V | | V | | V | V | | 8 | | 8 | 100 | |
| **17** | 3 | V | V | | X | | X | | X | | V | | V | | V | | V | V | | 7 | | 7 | 100 | |
| **18** | 2 | V | V | | X | | X | | X | | V | | V | | V | | V | V | | 7 | | 7 | 100 | |
| **19** | 5 | V | NA | | V | | X | | X | | V | | V | | V | | V | V | | 7 | | 8 | 86 | |
| **20** | 2 | V | V | | V | | X | | X | | V | | V | | V | | V | V | | 8 | | 8 | 100 | |
| **21** | 2.5 | V | V | | X | | X | | X | | V | | V | | V | | V | V | | 7 | | 7 | 100 | |
| **22** | 3 | V | V | | V | | X | | X | | V | | V | | V | | NA | V | | 7 | | 8 | 86 | |
| **23** | 5 | V | V | | V | | X | | X | | V | | V | | V | | V | V | | 8 | | 8 | 100 | |
| **24** | 4 | V | V | | V | | X | | X | | V | | V | | V | | V | V | | 8 | | 8 | 100 | |
| **25** | 6.5 | V | V | | V | | V | | X | | V | | V | | V | | V | V | | 9 | | 9 | 100 | |
| **26** | 4 | V | V | | V | | V | | X | | V | | V | | V | | V | V | | 9 | | 9 | 100 | |
| **27** | 6.5 | V | V | | V | | V | | X | | V | | V | | V | | V | V | | 9 | | 9 | 100 | |
| **28** | 4 | V | V | | V | | V | | X | | V | | V | | V | | V | V | | 9 | | 9 | 100 | |
| **29** | 6.5 | V | V | | V | | X | | X | | V | | V | | V | | V | V | | 8 | | 8 | 100 | |
| **30** | 3 | V | NA | | X | | X | | X | | V | | V | | V | | V | V | | 6 | | 7 | 83 | |
| **31** | 2 | V | V | | V | | V | | X | | V | | V | | V | | V | V | | 9 | | 9 | 100 | |
| **32** | 3.5 | V | NA | | X | | X | | X | | V | | V | | V | | V | V | | 6 | | 7 | 83 | |
| **33** | 6.5 | V | V | | V | | X | | X | | V | | V | | V | | V | V | | 8 | | 8 | 100 | |
| **34** | 6.5 | V | V | | V | | X | | X | | V | | V | | V | | V | V | | 8 | | 8 | 100 | |
| **35** | 5 | V | V | | V | | X | | X | | V | | V | | V | | V | V | | 8 | | 8 | 100 | |
| **36** | 4 | V | V | | V | | X | | X | | V | | V | | V | | NA | V | | 7 | | 8 | 86 | |
| **37** | 4 | V | V | | V | | X | | X | | V | | V | | V | | V | V | | 8 | | 8 | 100 | |
| **38** | 6 | V | V | | X | | X | | X | | V | | V | | V | | V | V | | 7 | | 7 | 100 | |
| **39** | 2.5 | V | V | | X | | X | | X | | V | | V | | V | | V | V | | 7 | | 7 | 100 | |
| **40** | 4 | V | V | | V | | X | | X | | V | | NA | | V | | V | V | | 7 | | 8 | 86 | |
| **41** | 4.5 | V | V | | V | | X | | X | | V | | V | | V | | V | V | | 8 | | 8 | 100 | |
| **42** | 5 | V | V | | V | | X | | X | | V | | V | | V | | V | V | | 8 | | 8 | 100 | |
| **43** | 4 | V | V | | V | | V | | X | | V | | V | | V | | V | V | | 9 | | 9 | 100 | |
| **44** | 3.5 | V | V | | X | | X | | X | | V | | V | | V | | V | V | | 7 | | 7 | 100 | |
| **45** | 6.5 | V | V | | X | | X | | X | | V | | V | | V | | V | V | | 7 | | 7 | 100 | |
| **# of surveys**  **filled out** |  | 45 | 40 | | 28 | | 9 | | 0 | | 45 | | 43 | | 44 | | 43 | 45 | | 342 | |  |  | |
| **# of surveys**  **sent** |  | 45 | | 45 | | 28 | | 10 | | 1 | | 45 | | 45 | | 45 | | | 45 | | 45 | 354 |  |  |
| **% of surveys filled out** |  | 100 | 89 | | 100 | | 90 | | 0 | | 100 | | 96 | | 98 | | 96 | 100 | |  | |  |  | |

### Fitbit Data

**Table A2.** Fitbit Inspire HR valid wear time during rehabilitation and at home at the individual level. The total valid wear time in minutes, and the number and percentage of valid wear days are presented for both phases, the phase in the rehabilitation clinic and the phase at home. A valid wear day was characterized by the wearing of the Fitbit for at least 10 hours during waking hours (6 a.m. to 11 p.m.). The day on which participants left the clinic and went back home was excluded.

| **IDs** | **EDSS** | **Total valid wear time during rehab [min]** | **Total valid wear days during rehab [n]** | **Total valid wear days during rehab [%]** | **Total valid wear time back home [min]** | **Total valid wear days back home [n]** | **Total valid wear days back home [%]** |
| --- | --- | --- | --- | --- | --- | --- | --- |
| **1** | 6 | 753 | 13 | 87 | 726 | 21 | 62 |
| **2** | 3.5 | 1392 | 24 | 100 | 1388 | 31 | 100 |
| **3** | 4.5 | 1356 | 38 | 97 | 1384 | 56 | 98 |
| **4** | 5.5 | 1355 | 18 | 100 | 1379 | 42 | 95 |
| **5** | 6 | 1377 | 19 | 100 | 1399 | 30 | 100 |
| **6** | 5.5 | 1395 | 25 | 100 | 1416 | 24 | 89 |
| **7** | 4 | 1375 | 33 | 100 | 1384 | 23 | 100 |
| **8** | 4 | 1402 | 16 | 100 | 1426 | 35 | 100 |
| **9** | 3.5 | 1394 | 17 | 100 | 1416 | 39 | 100 |
| **10** | 4.5 | 1341 | 18 | 100 | 1400 | 40 | 100 |
| **11** | 6 | 1399 | 24 | 100 | 1404 | 30 | 100 |
| **12** | 3.5 | 1343 | 24 | 96 | 1317 | 30 | 97 |
| **13** | 4 | 1322 | 16 | 100 | 1343 | 19 | 90 |
| **14** | 6.5 | 1396 | 18 | 100 | 1427 | 29 | 100 |
| **15** | 6 | 1330 | 22 | 100 | 1395 | 53 | 95 |
| **16** | 6 | 1349 | 18 | 100 | 1390 | 38 | 100 |
| **17** | 3 | 1385 | 19 | 100 | 1400 | 29 | 97 |
| **18** | 2 | 1402 | 17 | 100 | 1398 | 29 | 100 |
| **19** | 5 | 1333 | 18 | 100 | 1379 | 36 | 97 |
| **20** | 2 | 1408 | 25 | 100 | 1421 | 31 | 100 |
| **21** | 2.5 | 1328 | 18 | 100 | 1380 | 29 | 97 |
| **22** | 3 | 1410 | 21 | 100 | 1430 | 35 | 100 |
| **23** | 5 | 1388 | 22 | 100 | 1415 | 34 | 100 |
| **24** | 4 | 1429 | 18 | 95 | 1393 | 14 | 100 |
| **25** | 6.5 | 1413 | 29 | 100 | 1438 | 26 | 100 |
| **26** | 4 | 1431 | 27 | 96 | 1418 | 27 | 100 |
| **27** | 6.5 | 1340 | 27 | 96 | 1268 | 19 | 83 |
| **28** | 4 | 1396 | 30 | 100 | 1348 | 16 | 100 |
| **29** | 6.5 | 1402 | 25 | 100 | 1379 | 23 | 96 |
| **30** | 3 | 1377 | 15 | 100 | 1297 | 31 | 97 |
| **31** | 2 | 1418 | 31 | 100 | 1434 | 26 | 100 |
| **32** | 3.5 | 1396 | 13 | 93 | 1356 | 34 | 100 |
| **33** | 6.5 | 1388 | 18 | 100 | 1416 | 38 | 100 |
| **34** | 6.5 | 1376 | 23 | 92 | 1324 | 22 | 96 |
| **35** | 5 | 1255 | 23 | 100 | 1313 | 33 | 94 |
| **36** | 4 | 1380 | 24 | 100 | 1385 | 28 | 100 |
| **37** | 4 | 1397 | 24 | 100 | 1433 | 15 | 88 |
| **38** | 6 | 1386 | 15 | 100 | 1363 | 37 | 100 |
| **39** | 2.5 | 1218 | 15 | 100 | 883 | 30 | 97 |
| **40** | 4 | 1397 | 23 | 100 | 1435 | 28 | 97 |
| **41** | 4.5 | 1404 | 19 | 100 | 1378 | 32 | 91 |
| **42** | 5 | 1383 | 24 | 100 | 1412 | 31 | 97 |
| **43** | 4 | 1403 | 25 | 100 | 1399 | 21 | 100 |
| **44** | 3.5 | 1394 | 18 | 100 | 1431 | 39 | 100 |
| **45** | 6.5 | 1388 | 17 | 100 | 1379 | 30 | 100 |
| **Mean** |  | 1362 | 21 | 99 | 1362 | 30 | 97 |

## Study Participants

### Recruitment

**Table A3.** Reasons for non-participation in the study (n = 94).

| **Reasons for not participating in the study** | **N (%)** |
| --- | --- |
| **Inclusion criteria not met** | **n = 60** |
| EDSS <2 (or <3 but without walking  limitation) or >6.5 | 23 (38.3%) |
| Wheelchair use at home | 22 (36.7%) |
| Not technically affine | 7 (11.7%) |
| Cannot fill in the questionnaires in German | 3 (5.0%) |
| Too much cognitive impairment | 3 (5.0%) |
| Cannot download the Fitbit app | 2 (3.3%) |
| **Participation declined** | **n = 27** |
| Does not want to participate | 22 (81.5%) |
| Does not have time | 5 (18.5%) |
| **Other** | **n = 7** |
| No capacity for an additional participant | 7 (100%) |

### Study Participants’ Characteristics

**Table A4.** Comparison of some characteristics of the study population (n = 45), the persons assessed for eligibility but not enrolled in the study (referred as “non-participants” in the table; n = 94), and the study dropouts (n = 2). Non-participants manifest a high missing rate because data were not collected systematically among these persons.

| **Characteristics** | **Participants to the BarKA-MS study**  **(n = 45)** | **Non-participants to the BarKA-MS study (n = 94)** | **Dropouts**  **(n = 2)** |
| --- | --- | --- | --- |
| **Age**, median (IQR) | 46 (40-51) | 56 (48-63) | 36.5 (35-38) |
| Missing  information | 0 | 2 | 0 |
| **Sex** |  |  |  |
| Female | 29 (64.4%) | 57 (60.6%) |  |
| Male | 16 (35.6%) | 36 (38.3%) | 2 (100%) |
| Missing  information |  | 1 (1.1%) |  |
| **EDSS**, median  (IQR) | 4 (3.5-6) | 6 (5.0-6.5) | 3.8 (3.1-4.4) |
| Missing  information | 0 | 17 | 0 |
| **EDSS score** |  |  |  |
| 0 – 3.5 | 13 (28.9%) | 13 (13.8%) | 1 (50%) |
| 4 – 5.5 | 19 (42.2%) | 14 (14.9%) | 1 (50%) |
| ≥6 | 13 (28.9%) | 50 (53.2%) |  |
| Missing  information |  | 17 (18.1%) |  |

## Intervention Scalability Assessment Tool

**Table A5.** Questions from the Intervention Scalability Assessment Tool (34) used to assess the scalability of the BarKA-MS study.

| **Part A: „Setting the scene“**  The questions aim to describe „what is known about the current strategic/political/environment context“.  The purpose of this section is to outline the context for which the intervention is being considered for scale-up. |
| --- |
| 1. Is the problem of sufficient concern to warrant scale up of the intervention/program to address it? |
| 1. Will the outcomes delivered by this intervention address the needs of the target group (and/or) problem? |
| 1. Is addressing the problem consistent with policy/strategic directions or priorities? |
| 1. Will scaling up the intervention be strategically useful to funders/funding agency? |
| 1. Is there compelling evidence (from the literature or elsewhere) to indicate that the intervention is effective in addressing the problem in the target population? |
| 1. Is there evidence that the benefits of the intervention exceeded the costs? |
| **Part B: „Intervention implementation planning“ considers the potential implementation and scale-up requirements of the intervention within five domains.**  Readiness assessment questions explore „what might change from the current situation if the intervention is to be scaled up“.  The questions are designed to promote early thinking about potential implementation issues that would contribute to an intervention’s potential scalability. |
| **Domain B1: „Fidelity and adaption“** considers whether there are any proposed changes to the intervention required for scale up. |
| 1. Will the core components of the scaled up intervention be consistent with what was previously shown to be effective? |
| 1. If the core components of intervention are to be changed/adapted from its original form during scale up, will the impact of the changes/adaptations likely be favourable? |
| 1. Can program fidelity be monitored and/or maintained if implemented at scale? |
| **Domain B2: „Reach and acceptability“** considers the reach and acceptability of the intervention for the target population. |
| 1. Does the intervention have the potential to reach the intended target population at scale? |
| 1. Is the intervention likely to be acceptable to the target population? |
| **Domain B3: „Delivery setting and workforce“** considers the setting within which the intervention is delivered as well as the delivery workforce. |
| 1. Is the delivery setting(s) selected to deliver the program at scale consistent with that used in previous studies? |
| 1. Is the delivery workforce selected to deliver the program at scale consistent with that used in previous studies? |
| 1. Is the intervention likely to be acceptable to the delivery workforce involved in its delivery at scale? |
| 1. If the intervention requires integration into existing organisational or community structures, how likely is it to be feasible? |
| **B4: „Implementation infrastructure“** considers the potential implementation infrastructure required for scale up. |
| 1. Are the implementation infrastructure requirements of the intervention/program feasible for scale up? |
| **B5: „Sustainability“** considers the longer-term outcomes of the scale up, and how, once scaled up, the intervention could become sustainable over the medium to longer term. |
| 1. Is the level of integration of the intervention into delivery settings required for implementation at scale sustainable? |
| 1. Is the level of resourcing required to implement the intervention at scale sustainable? |
| 1. Is the delivery workforce selected for implementation at scale sustainable? |

## Devices Experiences

### During Rehabilitation


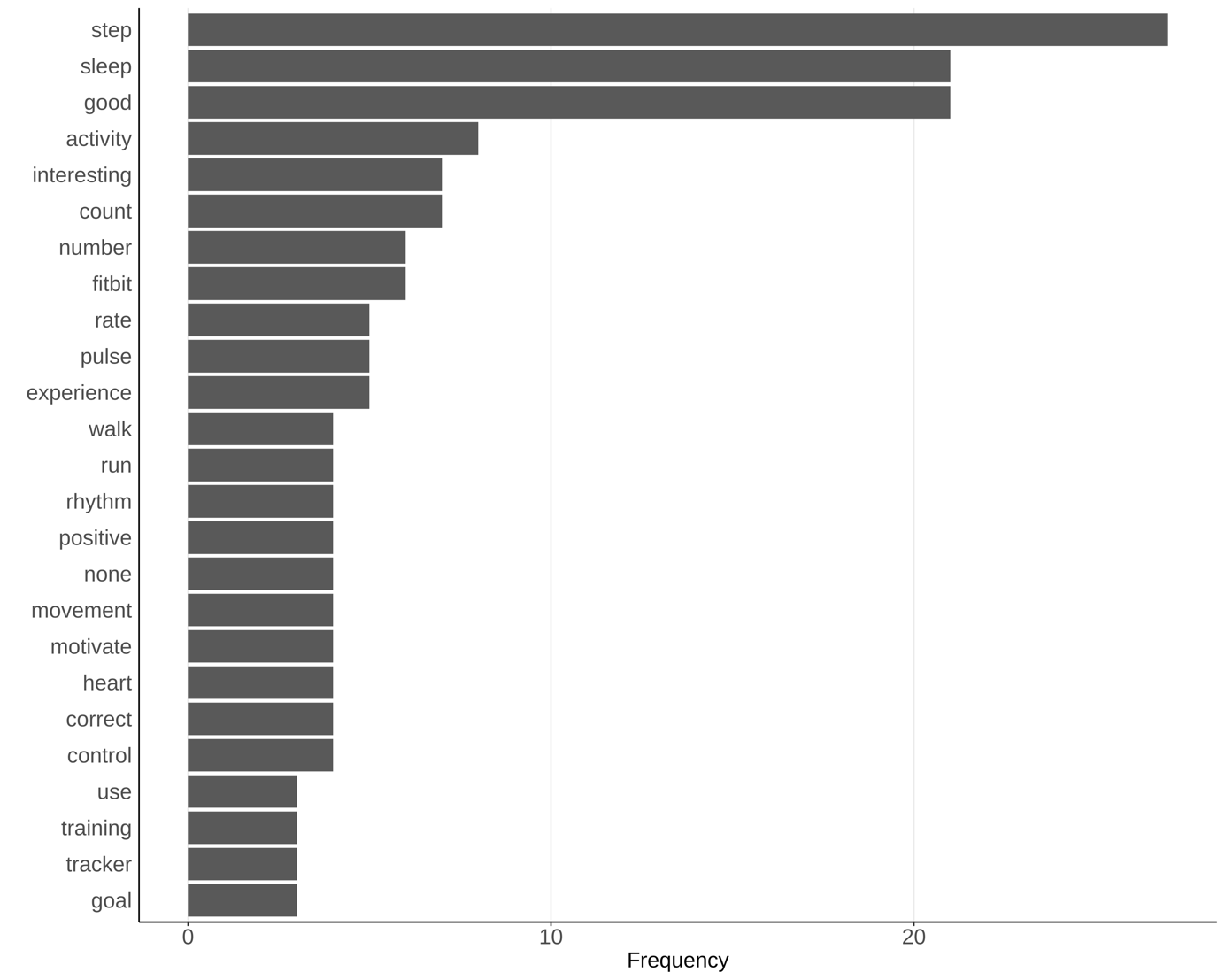


**Figure A2.** Words frequency of the 25 most common words in the answers (n = 107) to the question „What was your experience with activity trackers this week?“ asked during the rehabilitation phase. The word „none“ (position 16) mostly appeared alone as a finite answer and meant that there were not any new experiences.

### In Home Setting


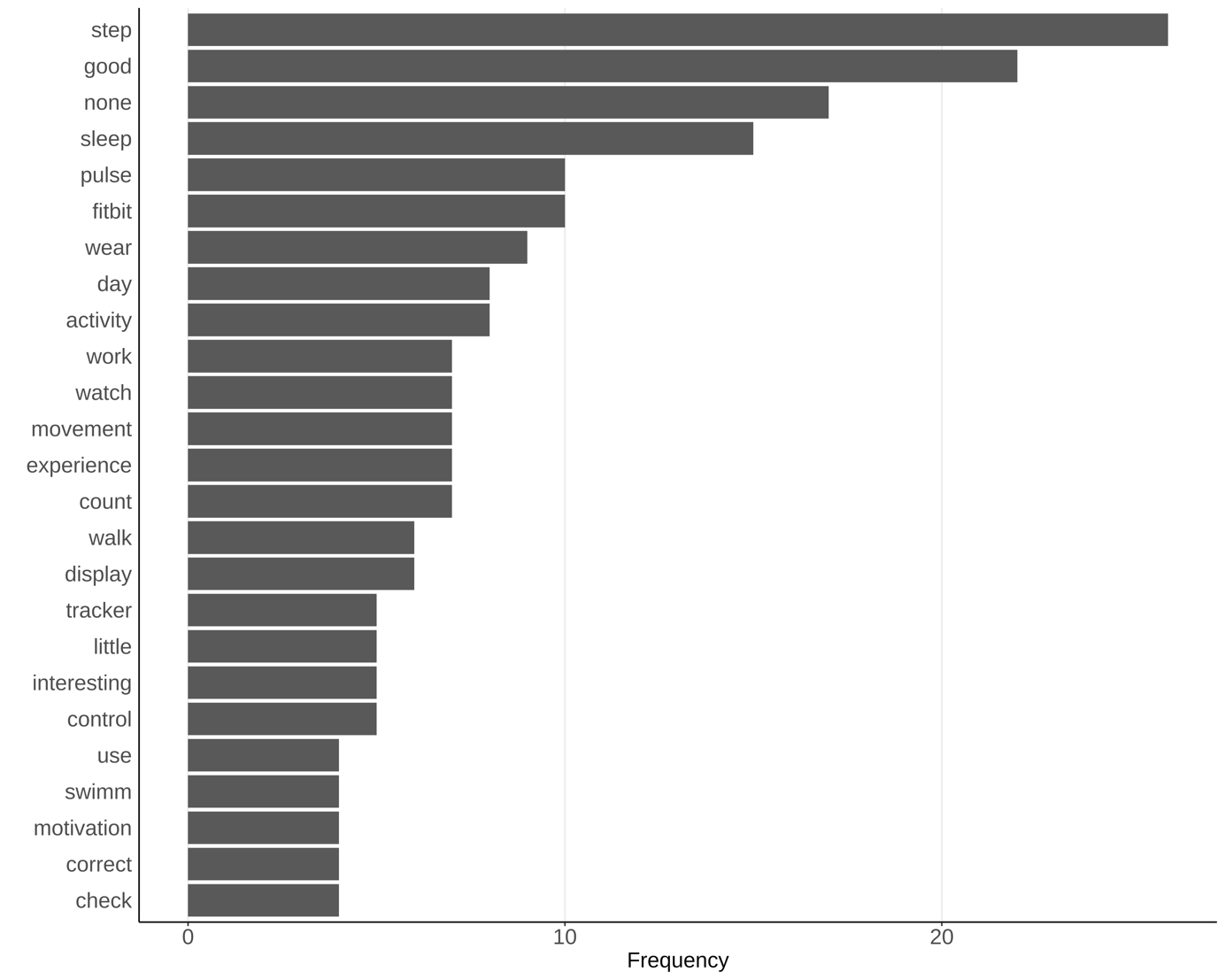


**Figure A3.** Words frequency of the 25 most common words in the answers (n = 142) to the question „What was your experience with activity trackers this week?“ asked during the home phase. The word „none“ (position 3) mostly appeared alone as a finite answer and meant that there were not any new experiences.

# References

1. Block VJ, Zhao C, Hollenbach JA, Olgin JE, Marcus GM, Pletcher MJ, et al. Validation of a consumer-grade activity monitor for continuous daily activity monitoring in individuals with multiple sclerosis. Mult Scler J - Exp Transl Clin. 2019 Oct 21;5(4):205521731988866.

2. Learmonth YC, Kinnett-Hopkins D, Rice IM, Dysterheft JL, Motl RW. Accelerometer output and its association with energy expenditure during manual wheelchair propulsion. Spinal Cord. 2016 Feb 1;54(2):110–4.

3. Motl RW, McAuley E, Snook EM, Scott JA. Validity of physical activity measures in ambulatory individuals with multiple sclerosis. Disabil Rehabil. 2006 Sep;28(18):1151–6.

4. Arend MG, Schäfer T. Statistical power in two-level models: A tutorial based on monte carlo simulation. Psychol Methods. 2019 Feb 1;24(1):1–19.

5. Casey B, Coote S, Donnelly A. Objective physical activity measurement in people with multiple sclerosis: a review of the literature. Disabil Rehabil Assist Technol. 2018 Feb 17;13(2):124–31.

6. Sasaki JE, Sandroff B, Bamman M, Motl RW. Motion sensors in multiple sclerosis: Narrative review and update of applications. Vol. 14, Expert Review of Medical Devices. NIH Public Access; 2017. p. 891–900.

7. Steinemann N, Kuhle J, Calabrese P, Kesselring J, Disanto G, Merkler D, et al. The Swiss Multiple Sclerosis Registry (SMSR): study protocol of a participatory, nationwide registry to promote epidemiological and patient-centered MS research. BMC Neurol. 2018 Aug 13;18(1):111.

8. Kurtzke JF. Rating neurologic impairment in multiple sclerosis: An expanded disability status scale (EDSS). Neurology. 1983;33(11):1444–52.

9. Sandroff BM, Riskin BJ, Agiovlasitis S, Motl RW. Accelerometer cut-points derived during over-ground walking in persons with mild, moderate, and severe multiple sclerosis. J Neurol Sci. 2014 May 15;340(1–2):50–7.

10. Becker H, Stuifbergen AK, Sands D. Development of a scale to measure barriers to health promotion activities among persons with disabilities. Am J Heal Promot. 1991 Jul 25;5(6):449–54.

11. Becker H, Stuifbergen A. What makes it so hard? Barriers to health promotion experienced by people with multiple sclerosis and polio. Fam Community Heal. 2004;27(1):75–85.

12. Kayes NM, McPherson KM, Schluter P, Taylor D, Leete M, Kolt GS. Exploring the facilitators and barriers to engagement in physical activity for people with multiple sclerosis. Disabil Rehabil. 2011 Jan 23;33(12):1043–53.

13. The IPAQ Group. International Physical Activity Questionnaire [Internet]. International Physical Activity Questionnaire. 2010 [cited 2022 May 11]. Available from: www.ipaq.ki.se

14. Craig CL, Marshall AL, Sjöström M, Bauman AE, Booth ML, Ainsworth BE, et al. International physical activity questionnaire: 12-Country reliability and validity. Med Sci Sports Exerc. 2003;35(8):1381–95.

15. Booth M. Assessment of physical activity: An international perspective. Res Q Exerc Sport. 2000;71:114–20.

16. Hobart JC, Riazi A, Lamping DL, Fitzpatrick R, Thompson AJ. Measuring the impact of MS on walking ability: The 12-item MS Walking Scale (MSWS-12). Neurology. 2003 Jan 14;60(1):31–6.

17. McGuigan C, Hutchinson M. Confirming the validity and responsiveness of the Multiple Sclerosis Walking Scale-12 (MSWS-12). Neurology. 2004 Jun 8;62(11):2103–5.

18. Penner IK, Raselli C, Stöcklin M, Opwis K, Kappos L, Calabrese P. The Fatigue Scale for Motor and Cognitive Functions (FSMC): Validation of a new instrument to assess multiple sclerosis-related fatigue. Mult Scler. 2009;15(12):1509–17.

19. Bandura A. Self-efficacy: The exercise of control. New York: W.H. Freeman and Company; 1997. 604 p.

20. Schwarzer R, Jerusalem M. Generalized Self-Efficacy scale. In J. Weinman, S. Wright, & M. Johnston, Measures in health psychology: A user’s portfolio. Causal and control beliefs. NFER-NELSON. Windsor, England; 1995. 35–37 p.

21. Kroenke K, Spitzer RL, Williams JBW. The PHQ-9: Validity of a brief depression severity measure. J Gen Intern Med. 2001;16(9):606–13.

22. Kroenke K, Strine TW, Spitzer RL, Williams JBW, Berry JT, Mokdad AH. The PHQ-8 as a measure of current depression in the general population. J Affect Disord. 2009 Apr 1;114(1–3):163–73.

23. Rabin R, De Charro F. EQ-5D: A measure of health status from the EuroQol Group. In: Annals of Medicine. 2001. p. 337–43.

24. Barin L, Salmen A, Disanto G, Babačić H, Calabrese P, Chan A, et al. The disease burden of Multiple Sclerosis from the individual and population perspective: Which symptoms matter most? Mult Scler Relat Disord. 2018 Oct 1;25:112–21.

25. Matter-Walstra K, Klingbiel D, Szucs T, Pestalozzi BC, Schwenkglenks M. Using the EuroQol EQ-5D in Swiss cancer patients, which value set should be applied? Pharmacoeconomics. 2014;32(6):591–9.

26. Perneger T V., Combescure C, Courvoisier DS. General population reference values for the french version of the euroqol EQ-5D health utility instrument. Value Heal. 2010 Jul 1;13(5):631–5.

27. Butland RJA, Pang J, Gross ER, Woodcock AA, Geddes DM. Two-, six-, and 12-minute walking tests in respiratory disease. Br Med J. 1982 May 5;284(6329):1607–8.

28. Enright PL. The Six-Minute Walk Test Introduction Standards and Indications 6-Minute Walk Test Versus Shuttle Walk Test Safety Variables Measured Conducting the Test Ensuring Quality Factors That Influence 6-Minute Walk Distance Interpreting the Results Improving the. Respir Care. 2003;48(8):783–5.

29. Severini G, Manca M, Ferraresi G, Caniatti LM, Cosma M, Baldasso F, et al. Evaluation of Clinical Gait Analysis parameters in patients affected by Multiple Sclerosis: Analysis of kinematics. Clin Biomech. 2017 Jun 1;45:1–8.

30. Watson MJ. Refining the ten-metre walking test for use with neurologically impaired people. Physiotherapy. 2002 Jul 1;88(7):386–97.

31. Paltamaa J, West H, Sarasoja T, Wikström J, Mälkiä E. Reliability of physical functioning measures in ambulatory subjects with MS. Physiother Res Int. 2005 Jun 1;10(2):93–109.

32. Richardson S. The Timed “Up & Go”: A Test of Basic Functional Mobility for Frail Elderly Persons. J Am Geriatr Soc. 1991 Feb 1;39(2):142–8.

33. Kalron A, Dolev M, Givon U. Further construct validity of the Timed Up-and-Go Test as a measure of ambulation in multiple sclerosis patients. Eur J Phys Rehabil Med. 2017;53(6):841–7.

34. Milat A, Lee K, Conte K, Grunseit A, Wolfenden L, Van Nassau F, et al. Intervention Scalability Assessment Tool: A decision support tool for health policy makers and implementers. Heal Res Policy Syst. 2020;18(1):1–17.
